# Targeting Fibrotic Scarring by Mechanoregulation of Il11ra1^+^/Itga11^+^ Fibroblast Patterning Promotes Axon Growth after Spinal Cord Injury

**DOI:** 10.1002/advs.202513476

**Published:** 2025-09-09

**Authors:** Longyou Xiao, Kaixi Shi, Wen Li, Jialin Liu, Pengfei Xie, Zijun Hu, Yu Dai, Haiyan Weng, Qiuju Yuan, Wutian Wu, Limin Rong, Liumin He

**Affiliations:** ^1^ Department of Spine Surgery The 3rd Affiliated Hospital, Sun Yat‐Sen University Guangzhou 510630 P. R. China; ^2^ Guangdong‐Hong Kong‐Macau Institute of CNS Regeneration (GHMICR) Jinan University Guangzhou 510632 P. R. China; ^3^ Department of Emergency The First Affiliated Hospital of Sun Yat‐sen University Guangzhou 510080 P. R. China; ^4^ Centre of Regenerative Medicine and Health, Hong Kong Institute of Science & Innovation Chinese Academy of Sciences Hong Kong 999077 P. R. China; ^5^ Re‐Stem Biotechnology Co., Ltd. Suzhou 215129 P. R. China

**Keywords:** aligned migration, fibrotic scarring, hydrogel mechanical strength, mechanotransduction

## Abstract

Fibrotic scarring remains a critic obstacle to axonal regeneration after spinal cord injury (SCI). Current strategies primarily concentrating on eliminating extracellular matrix (ECM) components neglect their dispensable roles in maintaining tissue integrity. Here, it is reported that the mechanical strength of an integrated hydrogel composed of hyaluronic acid‐graft‐dopamine and HRR peptide directs fibroblast migration, determining ECM deposition. The mechanical strength matching that of spinal cord induces fibroblast alignment, reshaping fibrotic scars into a parallel matrix, while the mechanical strength deviating from that of spinal cord fails to do so. Mechanical investigation identifies a previously unknown Il11ra1^+^/Itga11^+^ fibroblast subset that is specially associated with aligned infiltration and parallel ECM via mechanotransduction signaling cascade LRP6/β‐Catenin/MMP7, promoting axonal regeneration and boosting neural reconnections across the lesion. The study uncovers the mechanotransduction mechanism that remodels fibrosis progression through manipulating cellular components of fibrotic scars, providing novel insights into discovering potential therapeutic targets to resolve fibrosis after SCI.

## Graphical abstract

This study demonstrates that a hydrogel with mechanical properties matching the spinal cord directs fibroblast alignment, reshaping fibrotic scars into parallel matrices. A specific fibroblast subset mediates extracellular matrix remodeling through mechanotransduction, promoting axonal regeneration and neural reconnections, revealing potential therapeutic strategies to resolve fibrosis after spinal cord injury.

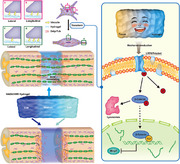

## 1 Introduction

The fibrotic scar, resulting from the excessive deposition and disorganization of the extracellular matrix (ECM), forms a critical barrier that hinders axonal regrowth after spinal cord injury (SCI).^[^ ^1^, ^2^ ^]^ The mainstream of current therapeutic treatments focuses on removing the fibrotic scar, such as degradation of ECM molecules,^[^ ^3^ ^]^ depleting scar‐forming fibroblasts ^[^ ^4^ ^]^ or inducing their migratory defects.^[^ ^5^ ^]^ However, the fibrotic scar plays an indispensable role in maintaining tissue integrity and sealing off the lesion.^[^ ^6^ ^]^ Moreover, fibrotic ECM serves as the scaffold to guide axonal regrowth based on integrin‐dependent axon‐substrate interactions.^[^ ^7^, ^8^ ^]^ ECM is essential for orchestrating the functions of cells and tissues, even under disease conditions.^[^ ^9^ ^]^ Therefore, it is arbitrary to categorize the fibrotic scar as a perceived inhibitor of neural regeneration considering its compositional complexity and context‐dependent functions. Thus, new insights into mechanisms governing fibrosis are imperative to discover new targets and develop strategies for SCI repair.

Since ECM proteins are secreted by resident cells, principally hypersecretory fibroblasts, cellular phenotypic and functional alterations in the aftermath of SCI play a critical role in fibrosis progression.^[^ ^1^ ^]^ In this context, approaches that focus on cellular responses induced by environmental cues instead of individual molecules may offer advantageous alternatives for deciphering fibrotic scarring mechanisms after SCI. The extent of ECM elimination has been not identified and is tricky to grasp. The potential of clinical translation is thus weak and not clear. New insights into mechanisms governing fibrosis are urgently needed to develop strategies for treating fibrotic scars. Recent advances highlight the evolvement of mechanotransduction pathways responding to fibrosis and the involvement in scarring, in which case mechanotransduction converts mechanical forces into biochemical stimuli and exerts impacts on multiple pathophysiological processes. Thus, the application of mechanomedicine concept is promising in the treatment of fibrosis from the viewpoint of mechanobiology.

Environmental mechanical strength serves as a critical biological mechanical cue to regulate cell proliferation, differentiation, and migration. It has been documented that cells tend to migrate toward substrates with increasing mechanical strength, a phenomenon well known as durotaxis.^[^ ^10^ ^]^ However, Isomursu et al. observed directed migration of glioma cells toward softer regions, which was defined as negative durotaxis.^[^ ^11^ ^]^ Despite progresses of mechanotransduction pathways in directing cell migration, the intricate evolution of intracellular signaling and the resultant downstream control of cell phenotype, as well as the association with matrix organization remain elusive. Addressing these questions helps to understand the mechanisms underlying scar formation, with an emphasis on the relationship between mechanotransduction pathways and their therapeutic implications.

Here in this study, we grafted an integrating hydrogel of different mechanical strength from hyaluronic acid‐*graft*‐dopamine (HADA) and the designer peptide HGF‐(RADA)_4_‐DGDRGDS (HRR) into SCI lesions ^[^ ^8^ ^]^ and investigated the impact of mechanotransduction on fibrosis following SCI. This study aims to uncover molecular mechanisms underlying the role of mechanotransduction in fibrosis and provide potential therapeutic targets for resolving fibrotic scars after SCI. Additionally, our study may also provide insights to the discovery of the potential therapeutic interventions to combat fibrosis and scarring.

## 2 Results

### 2.1 Fabrication of HADA/HRR Hydrogels with a Mechanical Strength Gradient

**Figure 1:**
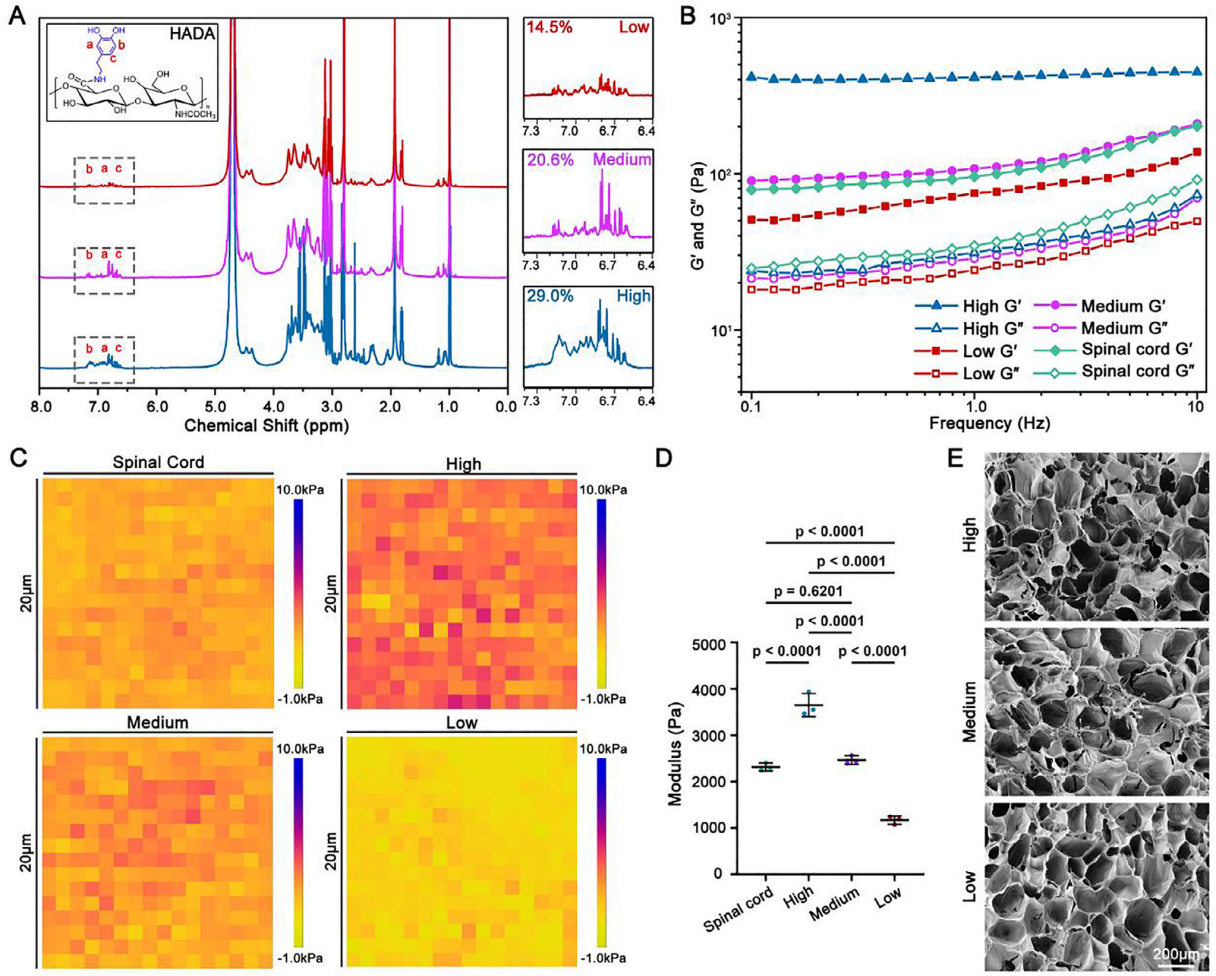
Preparation and characterization of HADA/HRR hydrogels with a mechanical strength gradient. A) ^1^H NMR spectra showing the successful synthesis of HADA with a mechanical strength gradient. B) Rheological measurements of High HADA/HRR hydrogel, Medium HADA/HRR hydrogel, Low HADA/HRR hydrogel, and rat spinal cord over an angular frequency range of 0.1 to 10 Hz. C,D) Representative elasticity heatmaps images C) and the quantification D) displaying the mechanical strength measured by atomic force microscopy (AFM) of the High HADA/HRR hydrogel, Medium HADA/HRR hydrogel, Low HADA/HRR hydrogel, and fresh rat spinal cord slices. N = 3 samples. E) Scanning electron microscopy (SEM) images of High HADA/HRR hydrogel, Medium HADA/HRR hydrogel and Low HADA/HRR hydrogel. Data were shown as the mean ± SD; *p* values were determined by two‐tailed one‐way ANOVA with Tukey's multiple‐comparisons test.

In order to investigate the impact of hydrogel mechanical strength on ECM organization after SCI, we first synthesized HADA with various dopamine (DA) grafting ratios to hyaluronic acid according to our previous study. DA was grafted onto HA to introduce catechol groups capable of undergoing oxidative coupling, thereby enabling spontaneous covalent crosslinking of HADA without the addition of xenobiotic cross‐linkers.^[^ ^8^ ^]^ HADA/HRR hydrogels were fabricated using the same composition ratio of HADA and HRR in the composite (**Figure** **1**A). A low DA grafting ratio of 14.5% generated HADA/HRR hydrogel with a G′ of 50 Pa as determined by rheological measurement (Figure 1B), which was designated as Low. 20.6% and 29.1% DA grafting ratio generated HADA/HRR hydrogel with G′ values of 100 and 400 Pa respectively, which was correspondingly designated as Medium and High. All three HADA variants with different DA grafting ratios were able to stably bind HRR. Considering the variation in the mechanical strength of spinal cord across the anatomical levels,^[^ ^12^ ^]^ we measured the rheological properties of rat thoracic segment (T9‐T10), which was the lesion site in the in vivo study. Interestingly, Medium exhibited mechanical strength similar to that of spinal cord (Figure 1B).

The mechanical strength of spinal cord and HADA/HRR hydrogels was further measured at a higher spatial resolution of nano‐to‐micro scales using atomic force microscopy (AFM), in which case single cell probes its surroundings with integrins.^[^ ^13^ ^]^ Elasticity heatmaps and corresponding quantification revealed that the elastic modulus of Medium was closely resembled of rat spinal cord (Figure 1C,D). Together, these results indicated that by adjusting DA grafting ratio, we fabricated HADA/HRR hydrogels with a mechanical strength gradient, and the Medium showed a mechanical strength comparable to that of rat spinal cord. Despite the mechanical strength variations, scanning electron microscopy (SEM) showed that the porous structure among HADA/HRR hydrogels was consistent (Figure 1E), which implicates the causal correlation between the mechanical strength of the hydrogels with the biological outcomes in subsequent studies.

### 2.2 HADA/HRR Medium Hydrogel Manipulated ECM Remodeling after SCI

**Figure 2:**
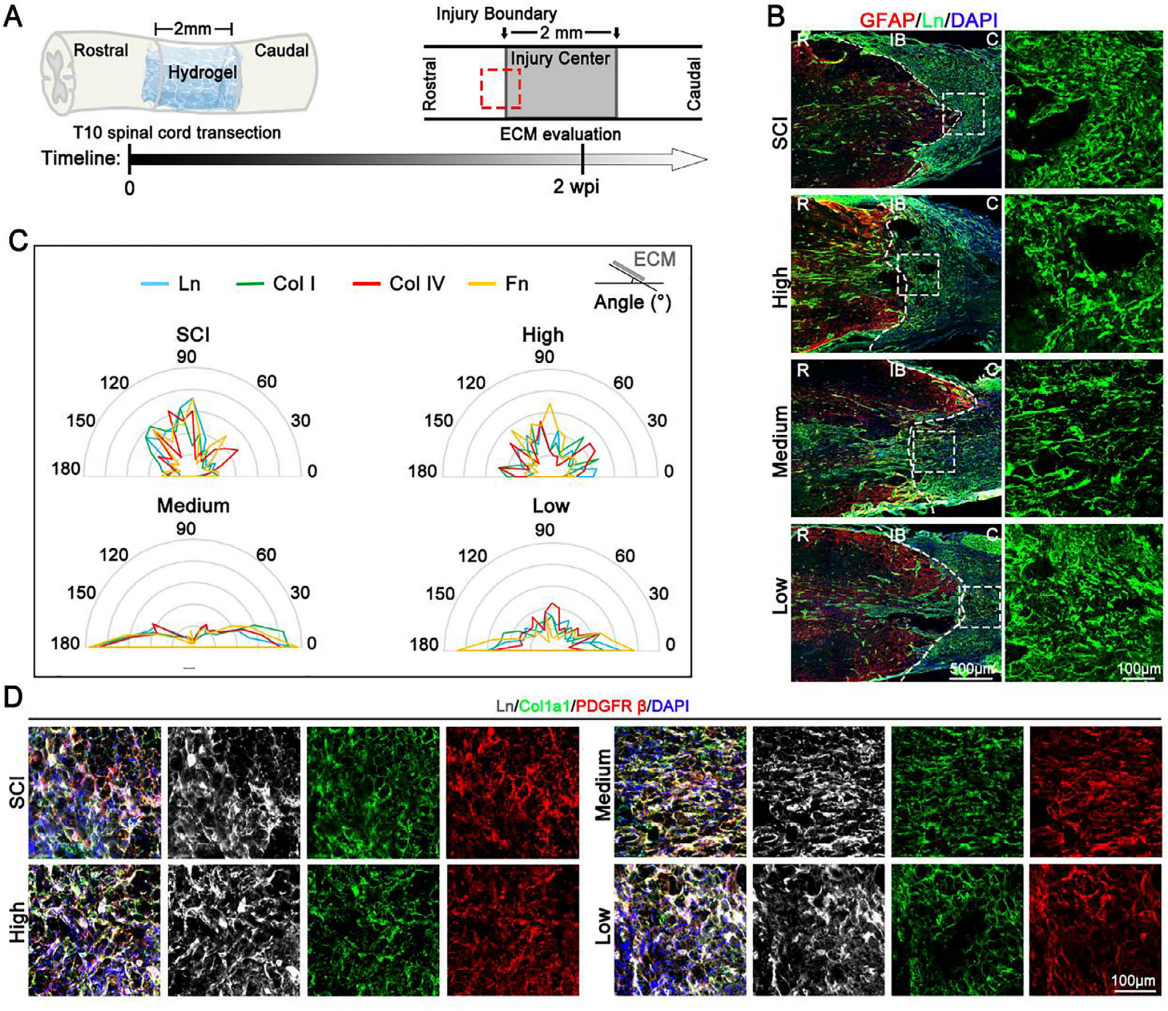
HADA/HRR Medium hydrogel manipulated ECM remodeling after SCI. A) Schematic of the experimental design. 2 mm of the rat spinal cord was transected and removed at the T10 segment and HADA/HRR hydrogels with High, medium and Low mechanical strength were grafted immediately after the SCI. 2 week after SCI (at 2 wpi, week post injury), the lesion region was collected for analysis. B) Representative immunostaining images showing the expression of glial fibrillary acidic protein (GFAP) and Laminin (Ln) along the longitudinal sections of spinal cord at 2 wpi. The boxed regions were shown at higher magnification to display the alignment of Ln (the middle rows). The alignment of Ln was color‐coded by OrientationJ (the right rows). Color scale bar represents the orientation of laminin along the longitudinal plane of spinal cord. White dashed lines indicate the injury boundary. R, rostral; IB, injury boundary; C, caudal. C) Statistical analysis of the orientations of extracellular matrix components (laminin, Ln; collagen I, Col I; collagen IV, Col IV; fibronectin, Fn) at the injury boundary, with angles measured relative to the horizontal axis as 0°. D) Representative immunostaining images showing the expression of Ln, Col1a1, and PDGFRβ at the injury boundary at 2 wpi.

We next investigated the formation and structure of fibrotic scars following the grafting of HADA/HRR hydrogels with different mechanical strength into the lesion after performing the transection at T10 segment (**Figure** **2**A). At 2 weeks post injury (wpi) dense and disorganized fibrotic scars were obviously detected (indicated by Laminin, Ln) at the lesion border of the injured spinal cord (Figure 2B). The fibrotic scars with a similar structure were observed in Low and High groups after SCI. However, Ln in the Medium group exhibited a parallel arrangement, which was in diametrical contrast to the disorganized fibrotic scars in other groups (Figure 2B). Quantitative analysis of Ln^+^ fiber orientation using the ImageJ plugin OrientationJ further confirmed that Ln fibers in the Medium group were oriented mainly along the longitudinal direction of spinal cord (Figure 2C). Additionally, other fibrotic ECM components, including collagen I (Col I), collagen IV (Col IV), and fibronectin (Fn), displayed a similar arrangement pattern depending on the mechanical strength of grafted hydrogels (Figure 2C; Figure S1A–C, Supporting Information). These findings suggest that grafting HADA/HRR hydrogel with mechanical strength similar to that of the native spinal cord into SCI lesion remodels the structure of fibrotic ECM from disorganized scars into an ordered arrangement along the longitudinal direction of spinal cord.

In agreement with our previous study,^[^ ^8^ ^]^ the infiltration of PDGFRβ^+^/Col1a1^+^ fibroblasts into the Ln^+^ matrix was observed across all groups (Figure 2D).

Together, the present results suggest that manipulating the mechanical strength of grafted hydrogel to a suitable mechanical strength comparable to native spinal cord can remodel the arrangement of fibrotic ECM, converting dense scars into parallel fibrous substrates. This remodeling of fibrotic ECM is not due to the changes of the infiltration of fibrotic scar‐forming cells. These findings motivated us to further explore the underlying mechanism.

### 2.3 Single‐Cell Profiling Identified an Itga11^+^/Il11ra1^+^ Fibroblast Subset Associated with Fibrotic ECM Remodeling

**Figure 3:**
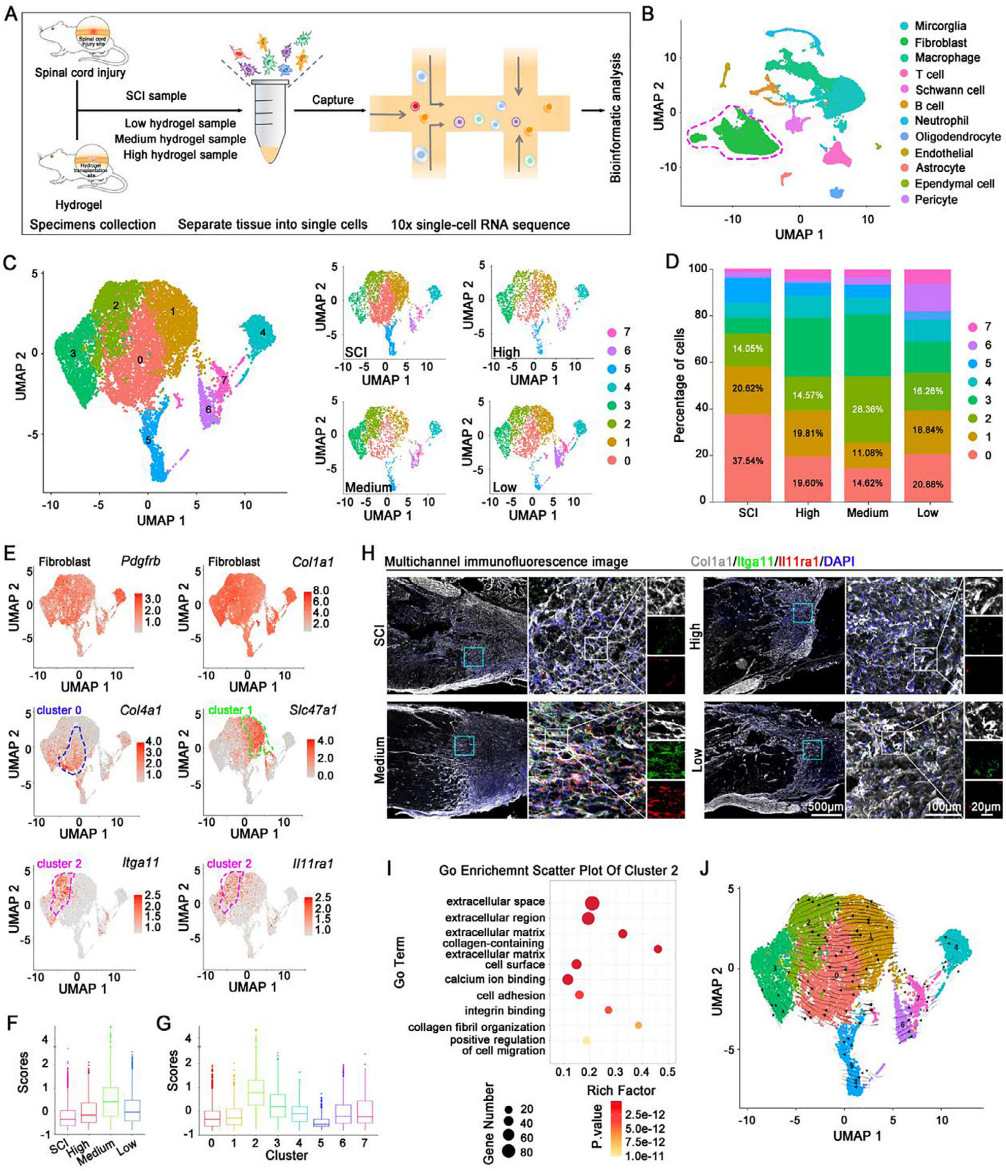
The Itga11^+^/Il11ra1^+^ fibroblast subset was associated with ECM remodeling. A) Schematic illustration of the experiment design of single‐cell RNA sequencing (scRNA‐seq). B) Uniform manifold approximation and projection (UMAP) plots showing cell components dissociated from the injured spinal cord tissue of different groups based on scRNA‐seq. C,D) UMAP plots C) and bar plot D) displaying the re‐clustering of fibroblasts from the injured spinal cord tissues in different groups based on scRNA‐seq. E) UMAP plots revealing the expression levels of *Pdgfrb*, *Col1a1*, *Col4a1*, *Slc47a1*, *Itga11*, and *Il11ra1* in fibroblast clusters based on scRNA‐seq. F,G) The module score showing the expression level of a specific gene module (based on *Itga11* and Il11ra1) within different experimental groups F) and fibroblast clusters G). H) Representative multichannel immunostaining images revealing the expression of Col1a1 in the longitudinal sections of spinal cord at 2 wpi. The blue boxed regions were higher magnifications revealing the expression of Col1a1, Itga11, and Il11ral, which were further enlarged in the white boxed regions with the separate channels. I) Gene Ontology (GO) enrichment scatter plot of cluster 2 fibroblasts. J) RNA velocity flow projected in the UMAP space of fibroblasts by dynamical model.

To provide detailed insights into the cellular feature within the fibrosis regions, single‐cell RNA‐seq (scRNA‐seq) was conducted at lesion borders 2 wpi (**Figure** **3**A). 12 cell types were identified using Unsupervised Uniform Manifold Approximation and Projection (UMAP) clustering, followed by further classification of specific cell types by canonical marker genes (Figure 3B; Figure S2A–C, Supporting Information). We focus on fibroblasts because they are directly associated with the production of fibrotic scar as the principal source.

Fibroblasts are a highly active cell type within fibrotic scars after SCI exhibit high heterogeneity in their origins, subsets and functions.^[^ ^14^, ^15^ ^]^ To reveal distinct fibroblast subclusters that contributed to ECM remodeling after grafting HADA/HRR Medium hydrogel, we re‐clustered fibroblasts based on their transcriptome profiles, and eight unique fibroblast clusters were identified (Figure 3C; Figure S3B, Supporting Information). Cluster 0–2 fibroblasts accounted for 72.21% of the total fibroblasts in the scar regions after SCI, suggesting that these subsets are major contributors to fibrotic scars (Figure 3D). The total proportion of cluster 0–2 fibroblasts significantly decreased to around 50% after grafting HADA/HRR hydrogels, despite the differences in mechanical strength. Compared with the Low and High groups, the proportion of cluster 2 increased in the Medium group while those of cluster 0 and 1 decreased (Figure 3D). Given the high phenotypic plasticity of fibroblasts, such findings indicate that hydrogel mechanical strength exerted impact on the dynamic transition of fibroblast subsets, and the Medium mechanical strength favored cluster 2.

Then we analyzed the detailed features of cluster 0–2 fibroblasts. All fibroblast clusters (cluster 0–7) expressed the typical fibroblast marker *Col1a1* and fibrotic scar‐forming fibroblast marker *Pdgfrb* (Figure 3E). Fibrosis‐related genes *Col4a1* and *Fn1* were enriched in Cluster 0 fibroblasts (Figure 3E; Figure S3C, Supporting Information), suggesting a close association with scar formation.^[^ ^16^ ^]^ Cluster 1 fibroblasts highly expressed meningeal fibroblasts markers *Slc47a1* and *Slc23a2* (Figure 3E; Figure S3D, Supporting Information), implying their origin.^[^ ^17^ ^]^ These identifications were further validated by the AddModuleScore algorithm (Figure S3E,F, Supporting Information). Cluster 2 fibroblasts that were specifically increased in the Medium group highly expressed cell membrane protein genes *Itga1l* and *Il11ra1*, which were accompanied with significant performance validated by the AddModuleScore algorithm (Figure 3E–G). Fluorescent staining revealed that Itga11 and Il11ra1 were predominantly expressed in the infiltrated fibroblasts when HADA/HRR Medium hydrogel was grafted after SCI. In contrast, few Itga11^+^/Il11ra1^+^ fibroblasts were detected in SCI, High and Low groups (Figure 3H), in which case dense and randomly distributed ECM was resulted. GO term analysis revealed that cluster 2 fibroblasts were enriched in pathways associated with ECM assembly (extracellular space, extracellular matrix) and migration (cell adhesion, integrin binding, and positive regulation of cell migration) (Figure 3I).

RNA velocity analysis indicated that cluster 2 mainly evolved from clusters 0 and 1 ^[^ ^18^ ^]^ (Figure 3J), which confirmed our analysis above. Cluster 2 subsequently developed into cluster 3, which was the adventitial fibroblast subpopulation documented to be associated with migration and proliferation (Figure S3B, Supporting Information).^[^ ^19^ ^]^ Overall, these findings indicate that grafting HADA/HRR hydrogel with the Medium mechanical strength modulates the conversion of fibrotic scar‐derived fibroblasts into specific Itga11^+^/Il11ra1^+^ fibroblast subset, which contributed to the remodeling of fibrotic ECM after SCI.

### 2.4 The Mechanical Strength of HADA/HRR Hydrogels Manipulated Fibroblast Migration Patterns

**Figure 4:**
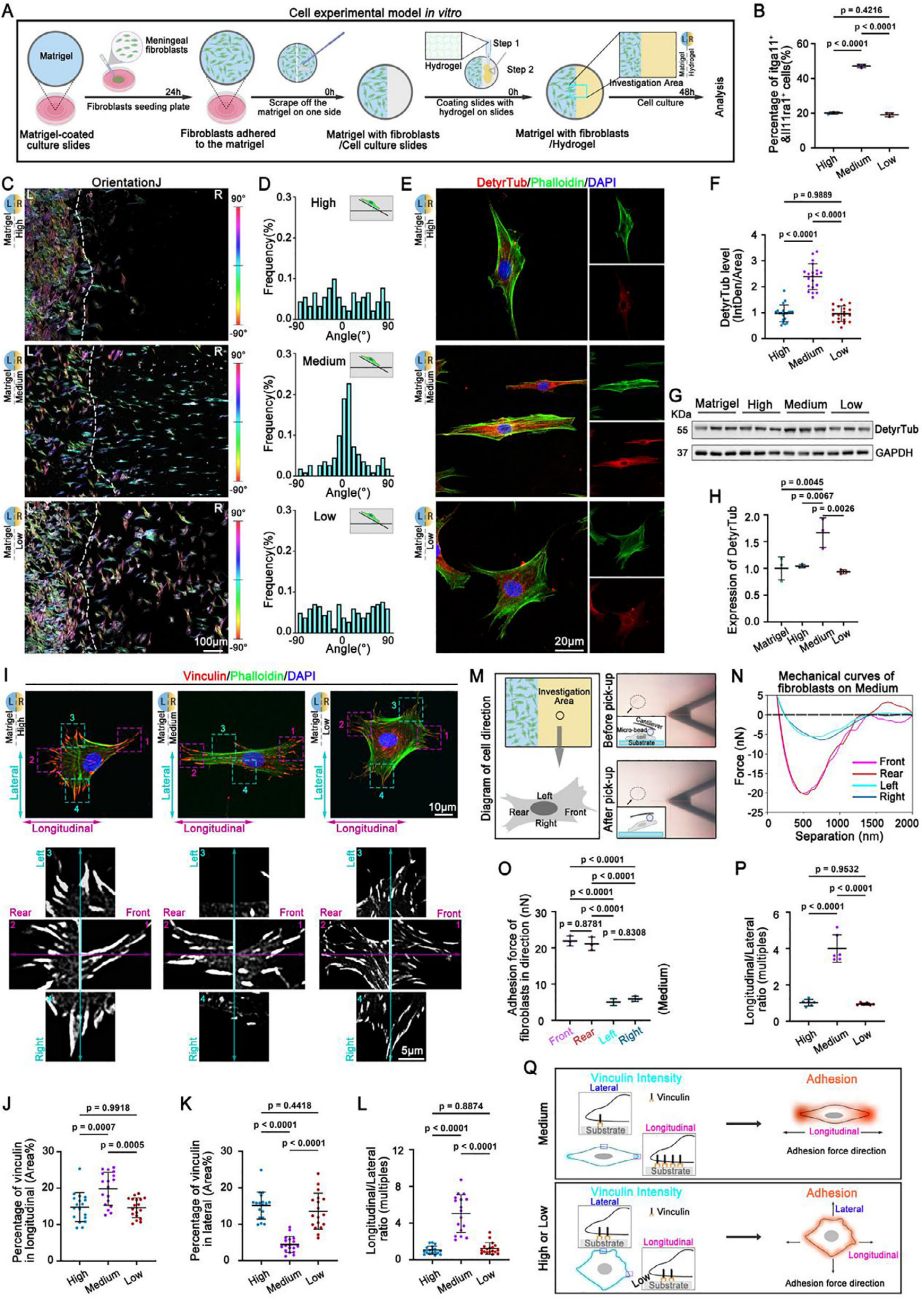
The mechanical strength of HADA/HRR hydrogels manipulated fibroblast migration patterns in vitro. A) Schematic of the experimental design to investigate the effect of mechanical strength gradient of the HADA/HRR hydrogel on the fibroblast migration in vitro. B) The quantification of flow cytometric analysis revealed the percentage of Itgall^+^/Il11ra1^+^ fibroblasts migrating in hydrogels with mechanical strength gradient in vitro. N = 3 samples. C) Representative images displaying the migration and the distribution of fibroblasts in different groups. The orientations of the fibroblasts were color‐coded using OrientationJ, which were indicated by the color scale bars. White dashed lines indicate the interface between the Matrigel and the hydrogel. L, left; R, right. D) The histogram represents the distribution of the orientations of the fibroblasts in each group. (E) Representative immunostaining images showing the expression of detyrosinated tubulin (DetyrTub) and phalloidin in the fibroblasts on the hydrogel side (right side) in different groups. F) The quantification of the relative fluorescence intensity of DetyrTub. N = 18 cells. G,H) Representative chopped Western blot images G) and quantitation H) displaying the expression of DetyrTub in the fibroblasts on the hydrogel side (the right side) with mechanical strength gradient, which migrated from the Matrigel side (the left side). N = 3 samples. I) Representative immunostaining images showing the expression pattern of vinculin and phalloidin in the fibroblasts on the hydrogel side (right side) in different groups, the boxed regions were the high magnification of vinculin signal. J–L) The quantification of the relative fluorescence intensity of vinculin in both the longitudinal J) and lateral K) orientations, as well as the longitudinal/lateral ratio L). N = 9 cells. M) Schematic illustrating the localization and positioning of detected fibroblasts using AFM. The representative images showing the cells before and after pick‐up are listed on the right. The index cell is indicated by the dashed circle and arrows. N) Mechanical curves of fibroblasts on Medium hydrogel in different directions generated by AFM. O) The statistical analysis of the adhesion force of the fibroblast in various directions on Medium hydrogel. N = 3 samples. P) The quantification of the longitudinal/lateral ratio of fibroblast adhesion force. N = 3 samples. Q) Schematic showing the relationship between vinculin intensity and adhesion force in fibroblasts migrating on the hydrogels with different mechanical strength. Data were shown as the mean ± SD; *p* values were determined by two‐tailed one‐way ANOVA with Tukey's multiple‐comparisons test.

The infiltrated Itga11^+^/Il11ra1^+^ fibroblasts were found to align well with the parallel fibrotic ECM along the longitudinal direction of spinal cord (Figure 3H). Thus, we wondered whether the mechanical strength of HADA/HRR hydrogels exerted any impact on the migration patterns of fibroblasts. To illuminate this, we isolated meningeal fibroblasts from rat spinal cord based on scRNA‐seq results mentioned above, and investigated their migration from Matrigel toward HADA/HRR hydrogels of different mechanical strength in vitro (**Figure** **4**A).

Consistent with in vivo results, flow cytometry analysis revealed that the percentage of Itga11^+^/Il11ra1^+^ fibroblasts migrating toward HADA/HRR Medium hydrogel significantly increased as compared to the other two groups (Figure 4B; Figure S4A,B, Supporting Information), indicating that the mechanical strength manipulated dynamic transition of fibroblast subsets autonomously during migration. Similar to previously documented negative durotaxis of glioma cells, we observed spinal cord meningeal fibroblasts were prone to migrate toward hydrogels with lower mechanical strength (Figure 4C). The increased cell number in the hydrogels with lower mechanical strength (both the Medium and Low groups) may not be attributed to the elevated cell proliferation, as the EdU incorporation experiment revealed that the cell division rates in Low and High groups was comparable (Figure S5A–C, Supporting Information). Interestingly, migrating fibroblasts on HADA/HRR Medium hydrogel showed a lower dividing rate than those on Low and High hydrogels, suggesting that hydrogel mechanical strength may also affect cell proliferation. According to AddModuleScore, cluster 2 (Itga11^+^/Il11ra1^+^) fibroblasts showed a low score of proliferation (Figure S5D–G, Supporting Information).

It was interesting that fibroblasts displayed a distinct migration pattern on HADA/HRR Medium hydrogel compared to those on the High and Low hydrogels (Figure 4C; Figure S6A, Supporting Information). Migrating fibroblasts with spreading morphologies were randomly distributed on HADA/HRR High and Low hydrogels, despite the difference in the number of migrating cells. On the contrary, migrating fibroblasts on HADA/HRR Medium hydrogel displayed polarized morphology along the longitudinal migration direction (Figure 4D; Figure S6A, Supporting Information). The polarization of migrating cells on HADA/HRR Medium hydrogel was further supported by the elevated expression of DetyrTub, an indicator of cell polarization (Figure 4E–H; Figure S6A, Supporting Information). The polarization of fibroblasts on HADA/HRR Medium hydrogel was associated with cell migration, as fibroblasts directly seeded on HADA/HRR Medium hydrogel displayed no evident cell polarization (Figure S6B–E, Supporting Information).

Cell polarization is generally associated with a specific arrangement and orientation of the cytoskeleton and adhesive complexes at cell membranes (e.g., focal adhesion, FA).^[^ ^20^ ^]^ We thus investigated the adhesion and spreading of migrating fibroblasts on HADA/HRR hydrogels. The vinculin‐positive FA plaques of cells migrating on HADA/HRR High and Low hydrogels exhibited vinculin plaques at the spreading periphery, whereas vinculin‐containing FAs were mainly localized at the front and rear regions on HADA/HRR Medium hydrogel (Figure 4I–L; Figure S7A, Supporting Information). The whole expression level of vinculin of cells on different hydrogels were comparable (Figure S7B,H,I, Supporting Information), suggesting that the hydrogel mechanical strength affected the distribution of FA proteins of the migrating fibroblasts rather than their total expression. Meanwhile, the distribution of FA proteins on HADA/HRR Medium hydrogel was associated with cell migration, as fibroblasts directly seeded on HADA/HRR Medium hydrogel displayed no evident correlation with the distribution of FA proteins (Figure S7C–G,J,K, Supporting Information).

The specialized arrangement of FA proteins generates tension in the direction to reinforce cell migration. To determine the adhesion force direction of migrating fibroblasts on HADA/HRR hydrogels, we measured adhesion forces in the longitudinal (front‐rear) and lateral (left‐right) directions of cellular peripherals using single‐cell force spectroscopy ^[^ ^2^, ^21^ ^]^ (Figure 4M). Similar detachment curves were observed among the four detected regions (front, rear, left, right) of fibroblasts migrating on HADA/HRR High and Low hydrogels, with negligible differences in the maximum detachment forces (≈10 nN) observed between these two hydrogels (Figure S8A–D, Supporting Information). However, divergent detachment curves were observed between the longitudinal (front and rear) regions and the lateral regions (left and right) of fibroblasts migrating on HADA/HRR Medium hydrogel (Figure 4N). Specifically, in contrast to the evenly distributed detachment forces of fibroblasts migrating on HADA/HRR High and Low hydrogel (Figure S8E,F, Supporting Information), the fibroblasts migrating on HADA/HRR Medium hydrogel showed larger detachment forces in longitudinal regions of than the lateral regions (21.50 ± 1.52 vs 5.50 ± 0.91nN, Figure 4O,P), indicating significant heterogeneity in cell adhesion.

The distribution of cell adhesion forces displayed a close correlation with that of FA structures (Figure S8G, Supporting Information). Together, these results indicate that the proper mechanical strength of hydrogels can guide cell migration by polarizing the distribution of FA proteins to generate adhesion forces along the direction of the migration, leading to longitudinal‐lateral asymmetry in the cytoskeleton (Figure 4Q).

### 2.5 Identification of MMP7 as a Key Factor in Regulating the Response of Fibroblasts to Environmental Mechanical Strength

**Figure 5:**
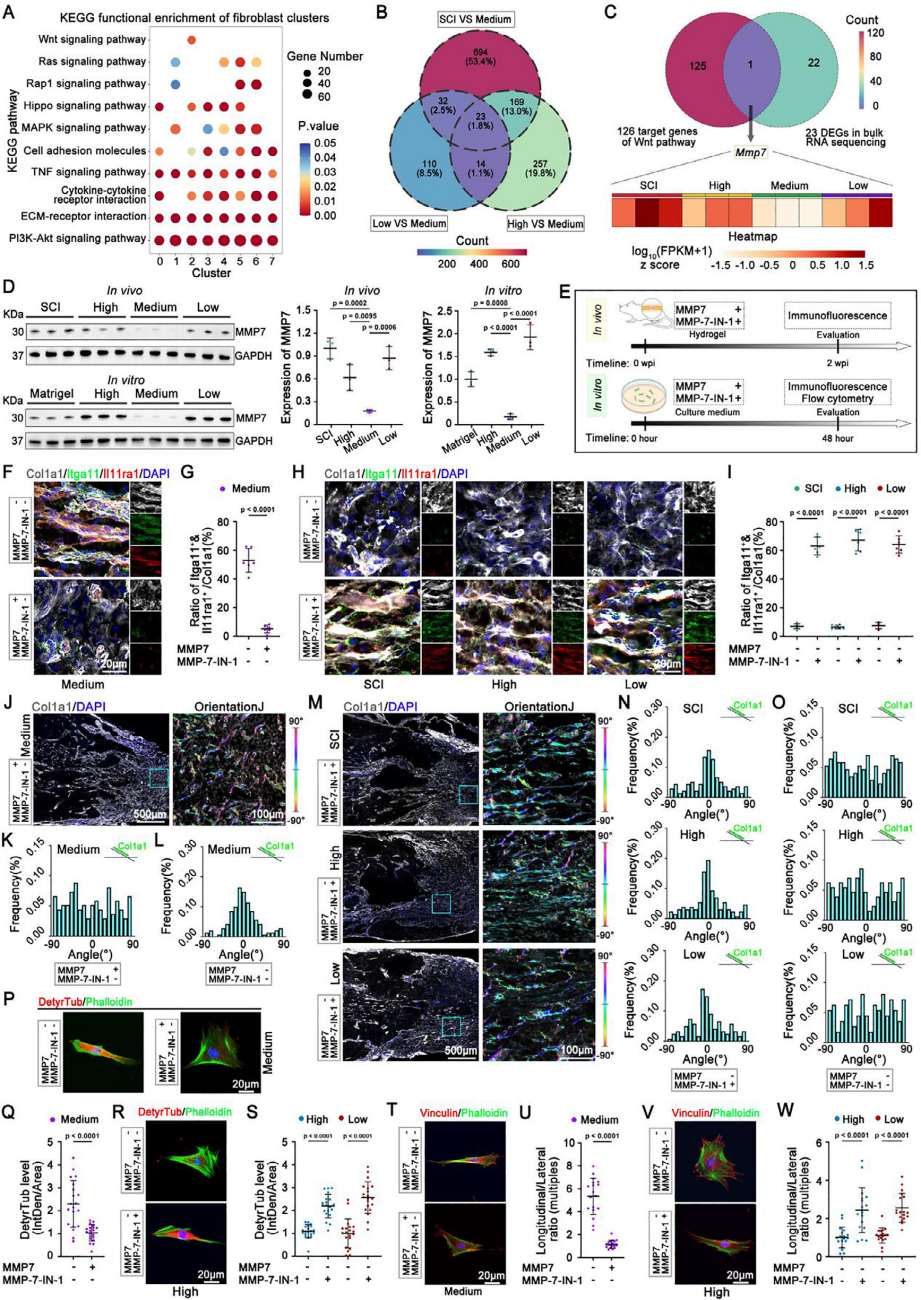
MMP7 was a key factor in regulating the response of fibroblasts to environmental mechanical strength. A) KEGG functional enrichment analysis of signaling pathways in fibroblast clusters based on scRNA‐seq. B) Venn diagram illustrated the relationships of the DEGs between the SCI and Medium groups, High and Medium groups, and Low and Medium groups from the Bulk RNA sequencing (bulk RNA‐seq). C) Venn diagram illustrated the overlapped DEG between the 23 DEGs in bulk RNA‐seq and 126 downstream effector molecules of the Wnt signaling pathway. The Heatmap showed the expression of *Mmp7* gene among samples based on bulk RNA‐seq. D) The representative chopped Western blot images and quantitation of the expression of MMP7 in vivo and in vitro, respectively. N = 3 samples, respectively. E) Schematic of the experimental design to investigate the role of MMP7 in vivo and in vitro. F) Representative multichannel immunostaining images showed the expression of Col1a1, Itga11, and Il11ral in the longitudinal sections of the spinal cord at 2 wpi following the intervention with MMP7 on Medium hydrogel group. The separate channels were presented in the right rows. G)The quantifications of the proportion of Itga11^+^/Il11ra1^+^ cells within the Col1a1^+^ population following the intervention with MMP7 on Medium hydrogel group. N = 6 animals. H) Representative multichannel immunostaining images showed the expression of Col1a1, Itga11, and Il11ral in the longitudinal sections of the spinal cord at 2 wpi following the intervention with MMP‐7‐IN‐1 on SCI, High hydrogel and Low hydrogel group, respectively. The separate channels were presented in the right rows. I) The quantifications of the proportion of Itga11^+^/Il11ra1^+^ cells within the Col1a1^+^ population following the intervention with MMP‐7‐IN‐1 on SCI, High hydrogel and Low hydrogel group. N = 6 animals. J) Representative multichannel immunostaining images revealed the expression of Col1a1 in the longitudinal sections of the spinal cord at 2 wpi following the intervention with MMP7 on Medium hydrogel group. The blue boxed regions were the higher magnifications of the color‐coded images revealing the orientation of Col1a1 using OrientationJ. The Color scale bars represented Col1a1 orientation. K,L) The quantifications of the orientations of Col1a1 following the intervention with MMP7 on Medium hydrogel group. M) Representative multichannel immunostaining images revealed the expression of Col1a1 in the longitudinal sections of the spinal cord at 2 wpi following the intervention with MMP‐7‐IN‐1 on SCI, High hydrogel and Low hydrogel group, respectively. The blue boxed regions were the higher magnifications of the color‐coded images revealing the orientation of Col1a1 using OrientationJ. The Color scale bars represented Col1a1 orientation. N,O) The quantifications of the orientations of Col1a1 following the intervention with MMP‐7‐IN‐1 on SCI, High hydrogel and Low hydrogel group, respectively. P) The representative images showing the co‐expression of DetyrTub and phalloidin in the fibroblasts that migrated on the hydrogel side (right side) following the intervention with MMP7 on Medium hydrogel group. Q) The quantification of the relative fluorescence intensity of DetyrTub following the intervention with MMP7 on Medium hydrogel group. N = 20 cells. R) The representative images showing the co‐expression of DetyrTub and phalloidin in the fibroblasts that migrated on the hydrogel side (right side) following the intervention with MMP‐7‐IN‐1 on High hydrogel group. S) The quantification of the relative fluorescence intensity of DetyrTub following the intervention with MMP‐7‐IN‐1 on High hydrogel group and Low hydrogel group, respectively. N = 20 cells. T) The representative images showing the co‐expression of vinculin and phalloidin in the fibroblasts that migrated on the hydrogel side (right side) following the intervention with MMP7 on Medium hydrogel group. U) The quantification of the relative fluorescence intensity of the vinculin with longitudinal/lateral ratio following the intervention with MMP7 on Medium hydrogel group. N = 9 cells. V) The representative images showing the co‐expression of vinculin and phalloidin in the fibroblasts that migrated on the hydrogel side (right side) following the intervention with MMP‐7‐IN‐1 on High hydrogel group. W) The quantification of the relative fluorescence intensity of the vinculin with longitudinal/lateral ratio following the intervention with MMP‐7‐IN‐1 on High hydrogel group and Low hydrogel group, respectively. N = 9 cells, respectively. Data were shown as the mean ± SD; *p* values were determined by two‐tailed one‐way ANOVA with Tukey's multiple‐comparisons test or two‐tailed paired *t*‐tests.

We then wondered how the mechanical strength of HADA/HRR hydrogel reconciles fibroblast heterogeneity with plasticity during fibrotic scarring. KEGG pathway was first conducted enrichment to analyze enriched pathways among fibroblast clusters and discovered great enrichment in pathways associated with cell mechanotransduction regulating proliferation, migration, and ECM assembly. Among these enriched pathways, Itga11^+^/Il11ra1^+^ (cluster 2) fibroblasts were specifically enriched in the canonical Wnt signaling pathway (**Figure** **5**A), which plays an important role in transmitting mechanical signaling in regulating cell fate by both activating and inactivating several signaling cascades.^[^ ^22^, ^23^ ^]^ To further identify the downstream effector molecules of the Wnt signaling pathway that were enriched in Itga11^+^/Il11ra1^+^ fibroblasts, bulk RNA‐seq was conducted 2 wpi to decipher genome‐wide transcriptome changes induced by the mechanical strength gradient of HADA/HRR hydrogels.

Pairwise comparisons of the transcriptional profile of the Medium group with the other three groups were performed. The heatmap and volcano plot revealed 918 differentially expressed genes (DEGs) between SCI and Medium group. Meanwhile, 463 DEGs and 179 DEGs were identified from Medium versus High and Medium versus Low comparisons, respectively. (Figure S9A–C, Supporting Information). Functional enrichment analysis revealed that these DEGs were enriched in ECM assembly and formation (Figure S9D–F, Supporting Information), suggesting a strong relationship between the mechanical strength gradient and ECM remodeling. The Venn diagram showed 23 key regulatory genes identified by intersecting DEGs from the three pairwise comparisons, which were the common feature genes specific to the medium mechanical strength (Figure 5B; Figure S9G, Supporting Information). One common effector, MMP7, was discovered when intersecting these 23 key DEGs with 126 target genes of the Wnt signaling pathway (Figure 5C).^[^ ^24^ ^]^ The downregulation of MMP7 was validated at both the transcriptional and translational levels in the Medium group compared with the other three groups in vivo (Figure 5C,D). MMP7 downregulation was also found in migrating fibroblasts on HADA/HRR Medium hydrogel in vitro (Figure 5D).

To assess the role of MMP7 in the induction of Itga11^+^/Il11ra1^+^ fibroblast subset during ECM remodeling, we supplemented MMP7 or MMP7‐IN‐1, a selective inhibitor of MMP‐7,^[^ ^25^ ^]^ into HADA/HRR hydrogels before injection into the lesion site in vivo, as well as into the culture medium in vitro (Figure 5E). The percentage of Itga11^+^/Il11ra1^+^ fibroblast subset decreased among fibroblasts in vivo (Figure 5F,G; Figure S10A,B, Supporting Information), and migrating fibroblasts on HADA/HRR Medium hydrogel in vitro (Figure S10E,F, Supporting Information) followed the supplementation of MMP7. On the contrary, the supplementation of MMP7‐IN‐1 increased the percentage of Itga11^+^/Il11ra1^+^ fibroblast subset after SCI as well as that of those grafted with HADA/HRR High and Low hydrogels in vivo (Figure 5H,I; Figure S10C,D, Supporting Information) and migrating fibroblasts on HADA/HRR High and Low hydrogels in vitro (Figure S10E,G, Supporting Information).

Consistent with the modulations of Itga11^+^/Il11ra1^+^ fibroblast subset, the supplementation of MMP7 disrupted the parallel arrangement of Col1a1 matrix at lesion border of spinal cord grafted with HADA/HRR Medium hydrogel (Figure 5J–L; Figure S10H,I, Supporting Information). It also abolished the polarization (indicated by the decreased level of DetyrTub, depolarized distribution of Vinculin and disturbed aligned migration) of the fibroblasts migrating on HADA/HRR Medium hydrogel in vitro (Figure 5P,Q, T,U; Figure S10L–N, S11A, C, S12A–E, K, Supporting Information). On the contrary, the supplementation of MMP7‐IN‐1 remodeled the disordered Col1a1 matrix into the parallel arrangement at the lesion border of spinal cord without grafting hydrogels (SCI) or grafted with HADA/HRR High and Low hydrogels (Figure 5M–O; Figure S10J,K, Supporting Information). The polarization (supported by the increased level of DetyrTub, polarized distribution of Vinculin and aligned migration) of the fibroblasts migrating on HADA/HRR Medium hydrogel was also increased in vitro (Figure 5R,S, V,W; Figure S10N–P, S11B,C,S12F–K, Supporting Information).

Taken together, these results indicate that suppressing MMP7 by the appropriate mechanical strength of hydrogel grafted after SCI plays a vital role in increasing the proportion of Itga11^+^/Il11ra1^+^ fibroblasts during fibrotic scarring, inducing the aligned migration/infiltration, and remodeling fibrotic scars into a parallel matrix.

### 2.6 Mechanotransduction Induced the Itga11^+^/Il11ra1^+^ Fibroblast Subset via LRP6‐Wnt/β‐Catenin‐MMP7 Signaling Axis

Mechanical properties of ECMs regulate essential cell behaviors, including differentiation, migration, and proliferation, through mechanotransduction. The evolution of intracellular mechanotransduction pathways converging on the nucleus to activate the downstream control of transcription and phenotypic expression remains elusive. The canonical Wnt/β‐catenin signaling pathway is an essential component of mechanically induced signal transduction related to cell activities and metabolism.^[^ ^23^ ^]^ Since the Wnt signal pathway and its effector MMP7 have been identified as a key factor in regulating the fibroblast composition in fibrotic scars in response to the mechanical loading of the ECMs, we then investigated the upstream components of Wnt signaling cascades involved in this process.

**Figure 6:**
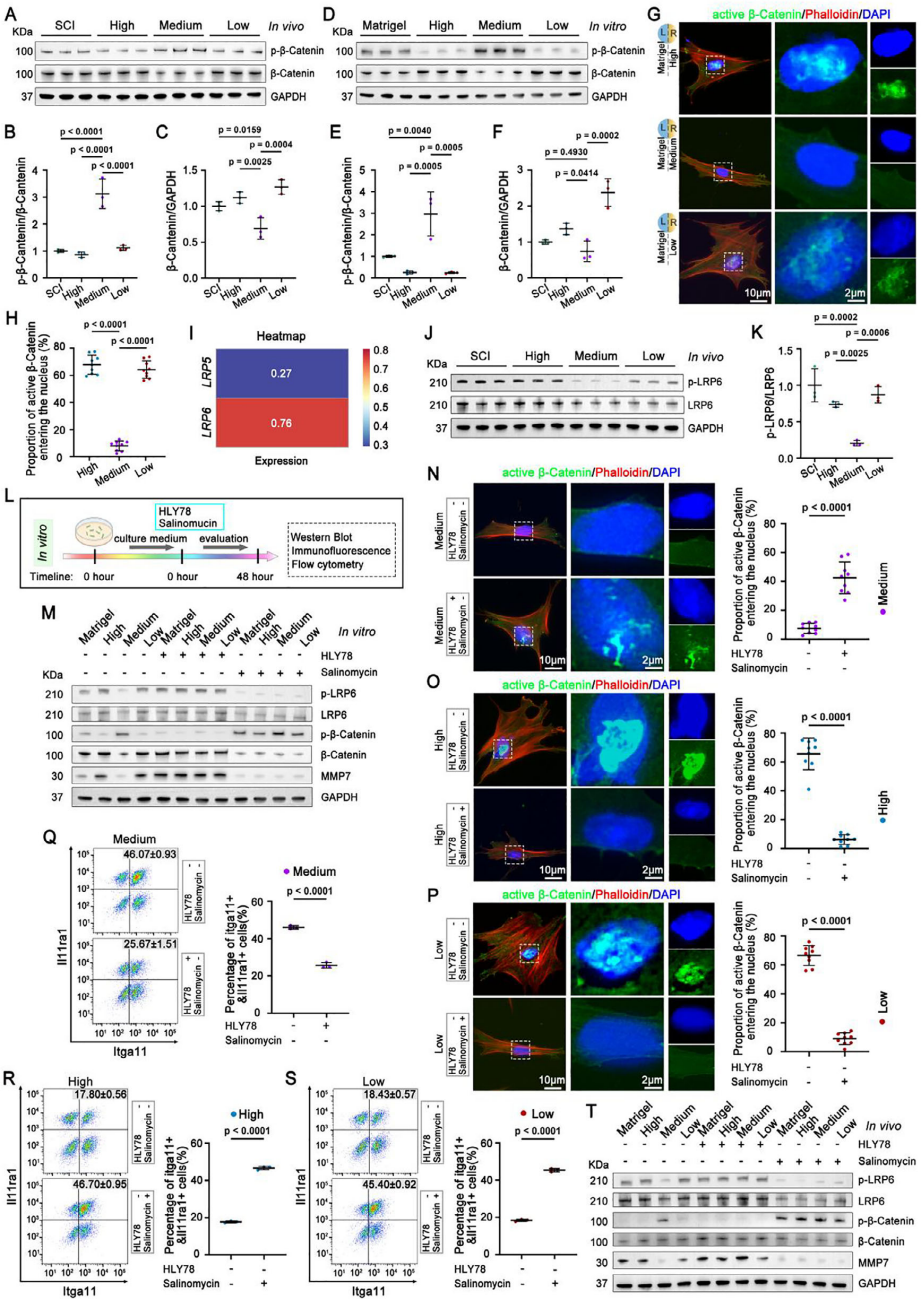
Mechanotransduction induced the Itga11^+^/Il11ra1^+^ fibroblast subset via LRP6‐Wnt/β‐Catenin‐MMP7 signaling axis. A–C) Representative chopped Western blot images A) and quantitation of the expression of phosphorylated β‐catenin B) and β‐catenin C) in vivo. N = 3 samples, respectively. D–F) Representative chopped Western blot images D) and quantitation of the expression of phosphorylated β‐catenin E), and β‐catenin F) in vitro. N = 3 samples, respectively. G) Representative multichannel immunostaining images revealing the expression active β‐catenin and phalloidin in the migrating fibroblasts in different groups. The white boxed regions were the high magnifications of the active β‐catenin signal and DAPI, which were presented in separate channels. H)The quantifications of the percentage of active β‐catenin entering nucleus. N = 9 cells. I) Heatmap showing the expression levels of *LRP5* and *LRP6* from scRNA‐seq. J,K) The representative chopped Western blot images J) and quantitation K) showing the expression of LRP6 and phosphorylated LRP6 proteins in vivo. N = 3 animals. L) Schematic of the experimental design for the in vitro validation. M) Western blot results of related proteins expressions in vitro following intervention with HLY78 and Salinomycin respectively. N) Representative multichannel immunostaining images showing the expression active β‐catenin and phalloidin in the migrating fibroblasts in Medium hydrogel group following intervention with HLY78. The white boxed regions were higher magnifications of the active β‐catenin signal and DAPI, which were presented in separate channels. The quantifications of the proportion of active β‐catenin entering nucleus following the intervention with HLY78 were listed on the right. N = 9 cells. O,P) Representative multichannel immunostaining images showing the expression active β‐catenin and phalloidin in the migrating fibroblasts in High hydrogel group O) and Low hydrogel group P) following intervention with Salinomycin. The white boxed regions were higher magnifications of the active β‐catenin signal and DAPI, which were presented in separate channels. The quantifications of the proportion of active β‐catenin entering nucleus following the intervention with HLY78 were listed on the right. N = 9 cells, respectively. Q) Flow cytometric analysis of Itgall^+^ Il11ra1^+^ fibroblasts in vitro in Medium hydrogel group following intervention with HLY78. N = 3 samples. R,S) Flow cytometric analysis of Itgall^+^ Il11ra1^+^ fibroblasts in vitro in High hydrogel group R) and Low hydrogel group S) following intervention with Salinomycin. N = 3 samples, respectively. T) Representative chopped Western blot images showing the expression of the related proteins in vivo following intervention with HLY78 and Salinomycin respectively. U) Schematic representation of the LRP6‐Wnt/β‐Catenin‐MMP7 pathway axis. Data were shown as the mean ± SD; *p* values were determined by two‐tailed one‐way ANOVA with Tukey's multiple‐comparisons test or two‐tailed paired *t*‐tests.

The activation of the canonical Wnt pathway is initiated when released Wnt proteins bind to receptor complexes, leading to increased β‐catenin levels in the cytoplasm and its nuclear translocation. On the other hand, β‐catenin is phosphorylated by GSK‐3β and degraded via the ubiquitin/proteasome pathway, resulting in the inactivation of the Wnt pathway.^[^ ^26^ ^]^ Treatment with HADA/HRR Medium hydrogel after SCI promoted the phosphorylation of β‐catenin, while decreased β‐catenin levels in vivo (**Figure** **6**A–C). The increase of the phosphorylated β‐catenin was also observed in the fibroblasts migrating on HADA/HRR Medium hydrogel in vitro, which was accompanied with the reduction of the expression of β‐catenin and its nuclear translocation (Figure 6D–H). Blocking the phosphorylation of β‐catenin using Laduviglusib elevated the levels of β‐catenin and MMP7 of fibroblasts migrating on HADA/HRR Medium hydrogel, whereas enhancing the phosphorylation of β‐catenin via (E)‐Ferulic acid attenuated the levels of β‐catenin and MMP7 of fibroblasts migrating on HADA/HRR High or Low hydrogels (Figure S13A,B, Supporting Information). The ultimate outcomes of the Wnt signaling are demonstrated to be genes with the activity controlled by β‐Catenin and T‐cell factor (TCF).^[^ ^27^ ^]^ In a study of human colorectal cancer, β‐Catenin interacted with TCF to regulate MMP7 expression.^[^ ^28^ ^]^ Accordingly, these results further support MMP7 as a direct downstream effector of the Wnt/β‐Catenin signaling pathway that regulates the mechanotransduction in the fibroblasts in response to the mechanical strength of HADA/HRR hydrogels.

Then we sorted the components located on cell membrane that sensed mechanical load and activated the downstream Wnt/β‐catenin cascade. Low‐density lipoprotein‐related receptor‐5 (LRP5) and‐6 (LRP6) is the coreceptor of the canonical Wnt pathways, which bind to the Frizzled (Fzd) to induce the nuclear translocation of β‐Catenin. LRP5 and LRP6 have been reported to regulate the mechanical loading‐induced bone formation.^[^ ^29^ ^]^ According to the scRNA‐seq data, the expression level of LRP6 was three times greater than that of LRP5 in the fibroblasts (Figure 6I). We thus focused on the role of LRP6 in mechanotransduction during the fibroblast filtration and the subsequent ECM deposition after SCI. We found a significantly decreased p‐LRP6/LRP6 ratio in the Medium group compared to the other three groups indicated of obviously lower phosphorylation of LRP6 in vivo (Figure 6J,K). A similar decrease in the p‐LRP6/LRP6 ratio was observed in the fibroblasts migrating on the HADA/HRR Medium hydrogels in vitro (Figure S13C,D, Supporting Information). These findings suggest that LRP6 in the fibroblasts can respond to the environmental mechanical strength gradient.

To further explore the potential role of LRP6 in mechanotransduction, we modulated the phosphorylation of LRP6 in fibroblasts migrating on the hydrogels in vitro by either HLY78, an activator of LRP6 phosphorylation, or Salinomycin, an inhibitor of LRP6 phosphorylation (Figure 6L). The enhancement of LRP6 phosphorylation of the Medium group decreased the levels of phosphorylated β‐Catenin, resulting in an elevated level of β‐catenin and its nuclear translocation (Figure 6M,N; Figure S13E,F, Supporting Information). As a consequence, the levels of MMP7 increased and the percentage of Itga11^+^/Il11ra1^+^ fibroblast subset reduced from 47.17 ± 1.01% to 25.67 ± 1.51% (Figure 6Q; Figure S13I,J, Supporting Information). Meanwhile, spreading cells were observed instead of spindle ‐shaped and polarized cells (Figure 6N). Conversely, suppression of LRP6 phosphorylation in fibroblasts migrating on HADA/HRR High and Low hydrogels resulted in increased levels of phosphorylated β‐Catenin, leading to decreased β‐Catenin expression and its nuclear translocation (Figure 6O,P; Figure S13G,H, Supporting Information). Meanwhile, the expression of MMP7 decreased, which resulted in an increased percentage of Itga11^+^/Il11ra1^+^ fibroblast subset (Figure 6M,R,S; Figure S13K, Supporting Information). Modulation of the phosphorylation of LRP6 also regulated β‐Catenin activity and MMP7 expression in vivo when HADA/HRR hydrogels were grafted onto the lesion (Figure 6T).

Collectively, these findings suggest that LRP6‐β‐Catenin‐MMP7 pathway cascade is involved in the mechanotransduction responsible for the specific Itga11^+^/Il11ra1^+^ fibroblast subset and their controlled infiltration by HADA/HRR Medium hydrogel after SCI. We thus proposed a mechanotransduction signaling axis involving in the manipulation of fibroblast compositions and fibrotic ECM assembly during the fibrotic scar formation after SCI: LRP6‐Wnt/β‐Catenin‐MMP7, where the modulation of LRP6 phosphorylation by environmental mechanical cues activates the Wnt pathway, inducing the accumulation of β‐Catenin in the cytoplasm and subsequently nucleus translocation to regulate MMP7 expression as a downstream target gene (Figure 6U).

### 2.7 Parallel Matrix Remolded by HADA/HRR Medium Hydrogel Guided Axonal Regrowth

**Figure 7:**
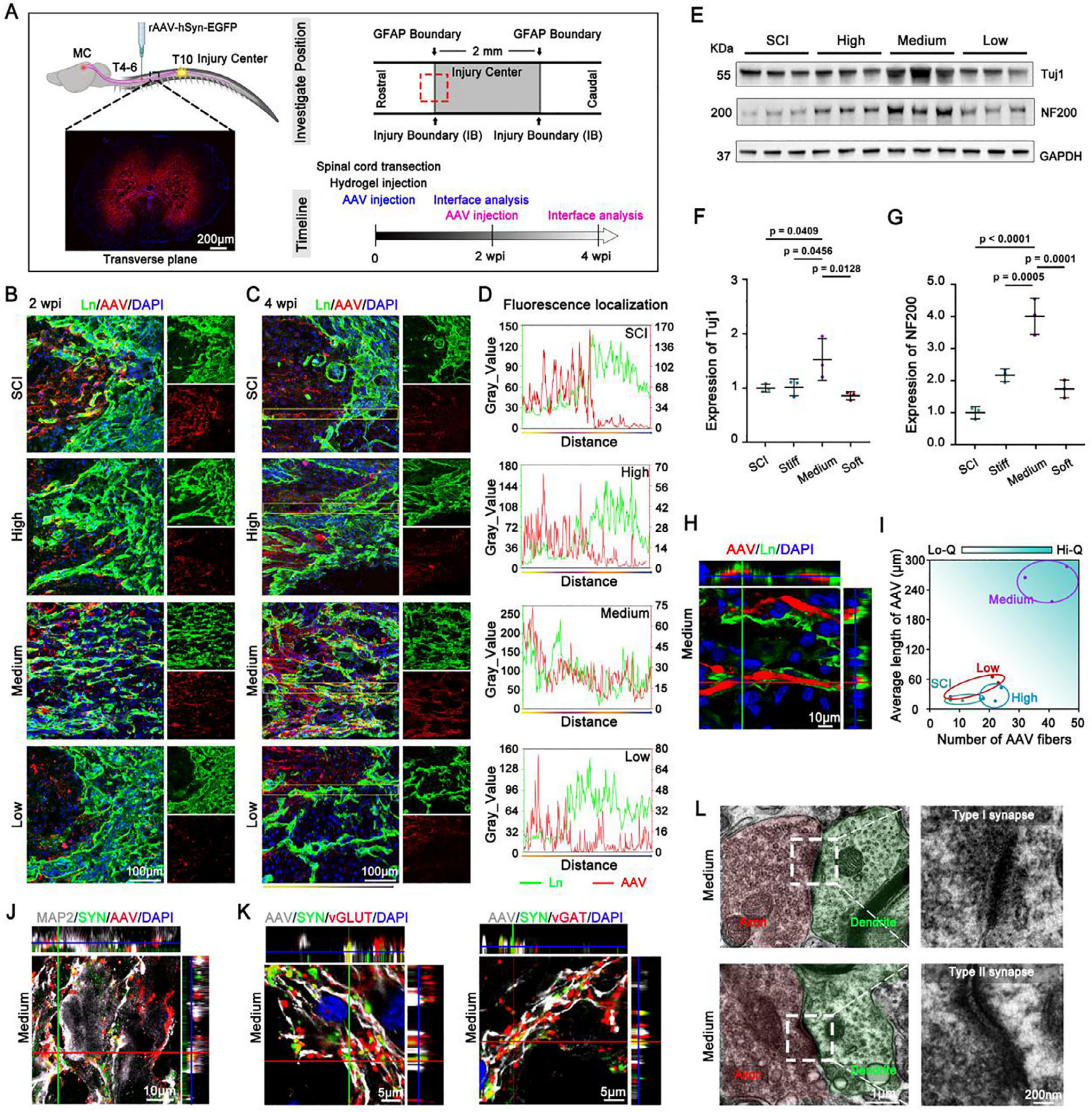
Parallel matrix remolded by HADA/HRR Medium hydrogel guided axonal regrowth. A) Schematic of the experimental design to investigate the axonal regeneration at the border of injury. B) Representative immunostaining images showing the expression of Ln and AAV along the longitudinal sections of spinal cord at the border of injury at 2 wpi. C) Representative immunostaining images showing the expression of Ln and AAV along the longitudinal sections of spinal cord at the border of injury at 4 wpi. The yellow boxes indicate the regions of interest to statistically analyze the co‐localization of AAV and Ln. D) The curves of analyze the co‐localization of AAV and Ln. E) Representative chopped Western blot images and quantifications F,G) of the expression of (βIII‐tubulin) Tuj1, and NF200 at 2 wpi. N = 3 animals, respectively. H) High‐ resolution confocal images of AAV and Ln at the injury boundary in the Medium hydrogel group at 4 wpi. I) Combined analysis of the quality of neural fibers based on number of AAV‐labeled fibers fibers and average length of AAV‐labeled fibers fibers. Hi‐Q, high quality; Lo‐Q, low quality. J) High‐ resolution confocal images of MAP2, SYN, and AAV in the injury boundary in the Medium hydrogel group at 4 wpi. K) High‐ resolution confocal images of co‐immunostaining of AAV, SYN, vGLUT, and AAV, SYN, vGAT respectively in the injury boundary in the Medium hydrogel group at 4 wpi. L) Representative transmission electron microscopy (TEM) images revealing the structure of type I synapse and type II synapse of at the injury center spinal cord tissue grafted with the Medium hydrogel at 4 wpi. The white box indicated an enlarged view of representative synaptic structures. Data were shown as the mean ± SD; *p* values were determined by two‐tailed one‐way ANOVA with Tukey's multiple‐comparisons test.

Promotion of axonal regrowth and the reestablishment of the disrupted neural connections for signal transduction are critical for the functional recovery after SCI. Since the mechanical strength of grafted hydrogels could remodel the disorganized fibrotic scars into a parallel matrix along the longitudinal axis of the spinal cord, we assessed whether such remodeled matrix facilitated axonal regrowth and neural connection reestablished. Retrograde tracing of neuronal axons in the thoracic spinal cord which were documented to contribute to walking production^[^ ^30^ ^]^ was employed to examine axonal regrowth 2 and 4 wpi, respectively (**Figure** **7**A).

Most of AAV‐labeled regenerating axons were observed at the borders of the Ln^+^ regions (Figure 7B) in the SCI, High, and Low groups 2 wpi with few axons detected within the Ln^+^ regions, indicating that axon regrowth ceased upon exposure to fibrotic scars. In contrast, robust axons regenerated into the Ln^+^ regions at the lesion border in the Medium group (Figure 7B). Meanwhile, Western blot analysis showed significantly promoted expression of βIII‐tubulin (Tubj) and NF200 in the Medium group compared to the other groups (Figure 7E–G).

After 4 weeks, regenerating axons were still blocked by the Ln^+^ regions in the SCI, High, and Low groups, whereas regenerating axons in the Medium group displaying a long, linear shape extended across the rostral border (Figure 7C,D). Images of higher magnification clearly revealed close association of regenerating axons with parallel Ln^+^ substratein the Medium group 4 wpi (Figure 7H). Quantitative analysis of the density and length of AAV‐labeled axons 4 wpi revealed that HADA/HRR Medium hydrogel significantly outperformed other groups in promoting axonal regeneration (Figure 7I). Meanwhile, a large number of NF200‐positive axons present at the site of injury in the HADA/HRR Medium hydrogel further validate axonal regeneration (Figure S14, Supporting Information).

Next, we investigated whether the regenerated axons promoted the formation of new neural connections across the lesion, and discovered abundant synaptic connections with the resident neurons at the lesion borders in the HADA/HRR group 4 wpi (Figure 7J). Both the excitatory neurotransmitter marker vesicular glutamate transporter (vGlut) and the inhibitory neurotransmitter marker vesicular GABA (γ‐aminobutyric acid) transporter (vGAT) were detected in the pre‐synaptic terminals of regenerated axons, suggesting the re‐establishment of neural connections between regenerated axons and resident neurons (Figure 7K). In addition, electron microscopy further supported the presence of asymmetrical type I synapses (representing excitatory synapses) with clear, rounded synaptic vesicles, and symmetrical type II synapses (representing inhibitory synapses) with uniform membrane densities ^[^ ^8^, ^31^ ^]^ (Figure 7L).

Collectively, these findings reveal the role of mechanical properties of HADA/HRR hydrogel to remodel the fibrotic ECMs at the border of spinal cord lesion, which guided axon regrowth into the lesion region and promoted the re‐establishment of heterogeneous neural connections across the lesion, potentially contributing to the reconstruction of spinal cord tracts.

## 3 Discussion

The fibrotic scar has emerged as a critical inhibitory factor for neural regeneration after SCI and there is no effective treatment available yet.^[^ ^32^ ^]^ In our latest publication, an integrating hydrogel fabricated from HADA/HRR showed self‐healing capacity, catechol‐mediated tissue adhesion, and biomechanical compatibility with spinal cord tissue. This hydrogel was able to induce the aligned infiltration of PDGFRβ^+^ fibroblasts at the lesion border after SCI, consequently creating a parallel fibrous matrix to guide axonal regrowth.^[^ ^8^ ^]^ In the present study, we provided evidence to reveal that mechanotransduction plays a key role in manipulating the controlled migration of a specific fibroblast Itga11^+^/Il11ra1^+^ subset via modulation of the canonical LRP6‐β‐Catenin‐MMP7 Wnt signaling cascade. So far, this is the first report that using artificial ECM to manipulate the cell composition of fibrotic scar‐forming fibroblasts, leading to a remodeled fibrotic matrix at the lesion border to facilitate axonal regrowth and neural connections after SCI.

Fibrosis occurs as a result of excessive deposition of ECM by fibroblasts. After SCI, two types of scars generated at the lesion region, glial scars consisting of astrocytes and fibrotic scars composed of ECM‐producing fibroblasts. Fibrotic scars are considered critical barriers to axonal regeneration, but complete ablation of fibrotic scars often leads to a larger cavity that is detrimental to tissue repair. Strategies to partially reduce scarring are promising to enhance axonal regeneration and functional recovery. However, the contradictory nature of ECM in physiological and pathological processes coupled with its intricate and context‐dependent functions persistently remains a notable challenge and laden with caveats.

Fibroblasts are a highly active cell type with a high functional heterogeneity in terms of cell subtypes and states, origins and fates.^[^ ^14^ ^]^ PDGFRβ has been reported as a marker of fibrotic scar‐forming fibroblasts after SCI. Our previous and current studies also found a high colocalization of fibrotic ECM signals with PDGFRβ. In addition, scRNA‐seq data confirmed that all fibroblast clusters analyzed in this study expressed high level of *Pdgfrb*. Grafting HADA/HRR hydrogels in the lesion significantly decreased the total ratio of cluster 0–2 fibroblasts, the major contributors to fibrotic scars after SCI. Particularly, for the first time, the Medium mechanical strength that was similar to the native spinal cord induced the conversion of fibrotic scar‐derived fibroblast subsets into specific Itga11^+^/Il11ra1^+^ fibroblast subset, which was identified through scRNA‐seq and bulk RNA‐seq, contributing to ECM remodeling after SCI.

Although notwithstanding significant progress in mechanotransduction has been made, there is very limited information on its impact on fibroblast plasticity and consequent physiological events so far. A recent study on bladder cancer (BC) identified a cancer‐associated PDGFRa^+^/ITGA11^+^ fibroblasts subset that created aligned ECM to assist BC cell lymphovascular invasion.^[^ ^33^ ^]^ Validation via in vitro investigation indicated that fibroblasts tended to migrate toward the HADA/HRR hydrogel with lower mechanical strength, undergoing negative durotaxis. However, only the Medium mechanical strength favored the Itga11^+^/Il11ra1^+^ fibroblast subset of a polarized state as determined by significant higher DetyrTub expression and the polarized distribution of adhesive complexes at cell membranes along the front‐rear orientation, in agreement with the controlled infiltration in vivo. Distinct FA and the distribution of cell adhesion force of migrating fibroblasts in response to hydrogel mechanical strength differences together indicated that the appropriate mechanical strength of hydrogels could guide cell migration by polarizing FA proteins distribution to generate adhesion forces along migration direction, leading to longitudinal‐lateral asymmetry in the cytoskeleton. These findings suggest that mechanotransduction may control the polarization and the aligned migration of Itga11^+^/Il11ra1^+^ fibroblasts in response to the mechanical strength of the hydrogels.

ECM is well known to provide architectural support for cell adhesion, and regulating cellular processes to maintain homeostasis in intact tissues.^[^ ^34^ ^]^ We here present a revolutionary innovation in manipulating fibroblast plasticity via mechanotransduction to convert fibrotic scars into a supportive substrate, conceptually distinct from traditional strategies focusing on removing resultant scars. We believe this approach could significantly sustain the development of therapeutic strategies for precisely treating fibrotic scars.

Finally, we conduct systematic in silico and wet lab experiments to comprehensively validate the results both in vivo and in vitro. A mechanotransduction signaling axis is proposed: LRP6‐Wnt/β‐Catenin‐MMP7. Through the modulation of MMP7 or LRP6 activity, we can manipulate the Itga11^+^/Il11ra1^+^ fibroblast subset within the fibrotic tissues in the lesion regions, accordingly regulating the migration patterns of fibroblasts and fibrotic ECM arrangement after SCI. Therefore, in addition to the traditional mechanotransduction paradigm, such as the widely studied Hippo/YAP/TAZ pathway,^[^ ^35^ ^]^ this study proposes the LRP6/Wnt/β‐Catenin/MMP7‐mediated mechanotransduction signaling axis to describe the precise molecular and physical mechanisms by which cells sense and respond to environmental mechanical properties. In this context, our study provides a specific, concrete overview that integrates extracellular mechanics with intercellular molecular cascades and active responses that govern cytoskeletal remodeling. To the best of our knowledge, our study, for the first time, identified LRP6/Wnt/β‐Catenin/MMP7 signaling cascade as a key role in ECM remodeling after SCI, providing potential targets to modulate the progress of fibrosis. We believe these findings provide an in‐depth mechanistic understanding of current mechanobiology, which would definitely integrate our physical perspective of mechanosensors with classical studies of molecular mechanotransduction.

Consequently, our study presents an entry point for understanding the crucial but elusive biomechanical cues in ECM remodeling, paving the way for exploring practical approaches to axonal regeneration after SCI in the coming years. Fibrotic scars have been proposed as a hallmark of diseases involving fibrosis progression. In line with this notion, mechano‐regulation can be expanded as a key target of ECM remodeling in treating these diseases.^[^ ^33^, ^36^ ^]^ Importantly, MMP7‐IN‐1, a selective inhibitor of MMP‐7, is identified to remodel disordered matrix into parallel arrangement, which definitely provide novel insights for potential therapeutic targets for fibrotic scarring, showing promising prospects of translational medicine.

### 3.1 The Limitation of the Study

In the current study, we identified mechanotransduction‐induced Wnt‐LRP6‐β‐Catenin‐MMP7 signaling as the key regulator to manipulate the dynamic transition of fibroblast subsets within the fibrotic scars and fibrotic ECM rearrangement after SCI. However, how Wnt‐LRP6‐β‐Catenin‐MMP7 signaling regulates the polarization of Itga11^+^/Il11ra1^+^ fibroblasts and controls their aligned migration has not been thoroughly elucidated. With the progress in empirically identifying mechanical signals that direct cell migration,^[^ ^11^, ^37^ ^]^ conceptual hypotheses have been proposed to explain the mechanisms of integrin‐mediated adhesions during cell migration, such as the putative “molecular clutch” ^[^ ^38^ ^]^ and “motor‐clutch” models.^[^ ^11^, ^39^ ^]^ However, our understanding of the dynamic evolution in cytobiology remains insufficient. Future work focusing on the relationship between the mechanotransduction‐induced Wnt‐LRP6‐β‐Catenin‐MMP7 signaling and cytoskeletal arrangement is required.

## 4 Conclusion

The presence of dense fibrotic scars has posed a significant challenge for axonal regrowth after SCI. In this study, we demonstrated that the mechanical strength of HADA/HRR hydrogel played a crucial role in ECM remodeling by manipulating the infiltration of PDGFRβ^+^ stromal cells. Notably, the HADA/HRR Medium hydrogel of a similar mechanical strength to native spinal cord induced a previously unknown Itga11^+^/Il11ra1^+^ fibroblast subset that contributed to the formation of parallel ECM. In line with this notion, mechano‐regulation could be the potential novel targets of ECM remodeling in the treatment of fibrosis after SCI or other diseases.

## 5 Experimental Section

### Materials

Hyaluronic acid (HA) was purchased from Toronto Research Chemicals INC. (Toronto, Canada). Dopamine hydrochloride (DA) was obtained from J&K Scientific Ltd. (Beijing, China). 1‐(3‐Dimethylaminopropyl)‐3‐ethylcarbodiimide hydrochloride (EDC) and N‐hydroxysuccinimide (NHS) were sourced from Meryer Chemical Technology Co., Ltd. (Shanghai, China). The self‐assembling peptide HRR was custom‐synthesized by Qiangyao Biotechnology Co., Ltd. (Hubei, China).

### Synthesis and Characterization of HADA

HADA was synthesized by conjugating dopamine to HA using EDC/NHS coupling. In brief, 758.6 mg HA (2 mmol) was dissolved in 120 mL deionized water, stirred at 35 °C under the nitrogen atmosphere. After 15 min, 690.5 mg NHS (6 mmol) and 1.1502 g EDC (6 mmol) were added, maintaining the pH between 5.0 and 5.5. Next, 189.6 mg DA (1 mmol) was introduced, and the reaction proceeded at 35 °C for 24 h in the dark. The mixture was then dialyzed against deionized water for 3 days and lyophilized to obtain HADA. The molar ratio of HA to DA in this reaction was 2:0.5, 2:1, and 2:1.75 in order to obtain HADA with different DA grafting rates.

### ^1^H NMR Spectroscopy

^1^H NMR spectra were recorded on a Bruker‐600 spectrometer (Bruker, Germany) at 600 MHz. Chemical shifts were expressed in parts per million (ppm) relative to deuteroxide as an internal standard. Samples were dissolved in deuterium oxide (D_2_O) and stirred at 25 °C to ensure homogenization.

### Fabrication of Hydrogels

HRR was dissolved in deionized water at 0.1% (w/v). Then HADA with the HA/DA molar ratio of 2:0.5, 2:1, and 2:1.75 were added into the HRR solution to reach a final total concentration of 2% (w/v). HADA has an average molecular weight of 168 kDa. The pH was adjusted to 7.0 using 1 m Tris. These composites were defined as “High”, “Medium”, and “Low”, respectively.

### Rheological Tests

The mechanical properties of the hydrogels and rat fresh spinal cord were assessed using a Kinexus Pro rheometer (Malvern Instruments, UK) with a 10 mm parallel plate geometry at 25 °C. A 300 µL sample was positioned between the plates with a 0.3 mm gap. Rheological measurements were conducted in Frequency Sweep mode from 0.1 to 10 Hz, with a shear strain of 0.5% and 10 samples per decade.

### Atomic force Microscope (AFM)

AFM analysis was performed using a Dimension FastScan AFM (Bruker, Germany) equipped with cantilevers featuring spherical tips to evaluate mechanical properties of the HADA/HRR hydrogels. The process began with bead gluing to the cantilevers to ensure precise measurements. Scanning was performed with a cantilever having a spring constant of 0.1 N m^−1^, with raster scans of 20 µm × 20 µm applied to the samples. Force‐distance curves were generated at each point within the scanned area. Hertz model was employed to analyze these curves for the elastic properties of the hydrogels.

### Scanning Electron Microscopy (SEM)

The structural morphology of HADA/HRR hydrogels was examined using an Ultra 55 Field Emission SEM (Zeiss, Germany) operating at an accelerating voltage of 5 kV. The samples were initially subjected to freezing at −20 °C and were cut with a scalpel in liquid nitrogen. These sections obtained were lyophilized to maintain their structural characteristics. Post‐lyophilization, the samples were mounted onto a copper stage. To enhance electron conductivity for SEM imaging, the mounted samples were coated with a thin layer of gold via sputter coating.

### Experimental Animals

Adult female Sprague‐Dawley (SD) rats, 7–8 weeks old and weighing 180–220 g, were obtained from the Animal Experimental Center at Sun Yat‐sen University. All procedures were approved by the Animal Ethics Committee of Sun Yat‐sen University (Approval Number: 2 022 000 677). The rats were housed under specific pathogen‐free conditions, with three animals per cage, maintained at 25 °C and a 12‐h light/dark cycle (7 am to 7 pm), with free access to food and water. The health of the rats, particularly post‐surgery, was monitored daily by both lab staff and researchers.

Postoperative care included daily intramuscular administration of ampicillin sodium (60 mg kg^−1^) for 7 days, along with oral ibuprofen (30–60 mg kg^−1^) to manage pain. During the acute phase following spinal cord injury (days 1–10 post‐injury), manual bladder expression was performed 2–3 times daily to prevent bladder overdistension and urinary tract infection, following established veterinary guidelines. Concurrently, abdominal massage was conducted for 10–15 min during each session to promote bowel function. Thereafter, the frequency was gradually reduced to once daily based on clinical assessment of residual urine volume and signs of autonomic recovery, as evaluated by trained personnel. The perineal area was kept clean and dry with frequent cleaning to maintain hygiene and prevent infection.

### Spinal Cord Injury Surgery

Anesthesia induction with 2% pentobarbital sodium (25 mg kg^−1^) was followed by maintenance with inhaled isoflurane (2%). A laminectomy at T10 level was performed using a #10 blade to incise the skin and muscle, followed by bone removal with rongeurs under a stereo microscope. After a vertical incision of the dura with a #11 blade and hemostasis, a 2 mm segment of the T10 spinal cord was excised with microscissors. The cavity was filled with 10 µL of HADA/HRR hydrogels via pipette. The dura was sutured with #10‐0 sutures, and the muscle and skin layers were closed with #4‐0 sutures. According to the experimental design, different groups received specific hydrogel treatments: the SCI group had a complete spinal cord transection with 2 mm without further treatment; the High group received HADA/HRR High hydrogel; the Medium group received HADA/HRR Medium hydrogel; and the Low group received HADA/HRR Low hydrogel.

### Immunofluorescence Staining

For animals, procedures were performed in full compliance with the ethical guidelines established by the Animal Ethics Committee of Sun Yat‐sen University. Prior to any surgical procedure, rats were anesthetized using an intraperitoneal injection of sodium pentobarbital (25 mg kg^−1^). To ensure sufficient depth of anesthesia for the surgical phase, isoflurane (2% in oxygen) was administered for maintenance throughout the duration of the procedure. For humane euthanasia prior to thoracotomy and perfusion, a terminal dose of sodium pentobarbital (150 mg kg^−1^, intraperitoneal) was administered. Death was confirmed by the absence of spontaneous breathing, heartbeat, and pupillary light reflex, ensuring that no invasive procedures were conducted on live animals. Cardiac perfusion was performed with 0.9% saline, followed by 4% paraformaldehyde (PFA, YOBIBIO, China) for tissue fixation. Tissue adjacent to the spinal cord injury was excised and fixed in 4% PFA at 4 °C for 24 h. Post‐fixation, tissues were cryoprotected with a gradient of sucrose solutions (10%, 20%, and 30%) in 0.1 m phosphate‐buffered saline (PBS, Beyotime, China) and embedded in OCT compound before rapid freezing. Samples were stored at −80 °C until sectioning. Longitudinal sections (12 µm) were cut with a cryomicrotome (Leica CM1950, Leica Microsystems, Germany) and mounted on slides. Immunofluorescence staining involved rinsing sections with PBS (3× 5 min) to remove OCT, followed by permeabilization with 0.1% Triton X‐100 (Beyotime, China) in PBS for 10 min. Blocking was performed with 5% bovine serum albumin (BSA, Biofroxx, Germany) in PBS for 1 h at 25 °C. Primary antibodies were diluted in 5% BSA/PBS and incubated overnight at 4 °C (Table S1, Supporting Information). After washing with PBS (3 × 5 min), sections were incubated with fluorophore‐conjugated secondary antibodies in PBS for 1 h at 25 °C in the dark. Sections were washed again (3 × 10 min) and mounted with DAPI‐containing Mounting Medium (Abcam, UK). Fluorescent images were acquired with an Axio Imager Z2 microscope (Zeiss, Germany) and a Leica STELLARIS STED confocal microscope (Leica Microsystems, Germany) for high‐resolution imaging.

At the designated time points, the cell culture medium in vitro was removed, and the cells were washed three times with PBS. Cells were fixed with 4% PFA at 25 °C for 20 min. After being washed twice with PBS, cells were permeabilized with 0.5% Triton X‐100 in PBS at 25 °C for 15 min. The immunofluorescence staining was conducted as described above, including blocking, primary antibody incubation, secondary antibody incubation, and imaging.

For histological analysis, spinal cord tissues were harvested and transversely cryosectioned into approximately 50 serial sections per sample, with each section consecutively numbered. To ensure consistent and unbiased sampling across animals and groups, every 15th section was selected for downstream analysis, yielding three non‐adjacent representative sections per animal. Regions of interest were defined based on lesion center and perilesional boundaries using anatomical landmarks. For each selected section, high‐resolution images were acquired under identical exposure settings across groups. Quantification was performed within standardized fields using ImageJ, and signal intensity or positive cell counts were normalized to the analyzed area. All analyses were conducted in a blinded manner.

### Angle Statistical Analysis

For ECM and fibroblast distribution analysis both in vitro and in vivo, three distinct 0.5 mm × 0.5 mm areas were selected from each section for detailed examination. The orientation of the target relative to the horizontal axis was measured using ImageJ software (v1.53). Quantitative analysis evaluated the alignment of ECM or fibroblasts within the designated regions. The results were presented as pie charts and frequency distribution histograms.

### Single‐Cell Library Preparation and Sequencing

Animals were deeply anesthetized and euthanized prior to perfusion with ice‐cold 0.9% saline. Fresh samples (≈5 mm in length) were harvested from injury sites and rinsed with ice‐cold PBS, which were then cut into 2 mm pieces and washed three times with ice‐cold PBS. Tissue fragments were enzymatically digested in a solution containing 2 mg mL^−1^ papain (Worthington Biochemical Corporation) and 0.005% DNase I (Sigma‐Aldrich) prepared in Hibernate‐A medium (Thermo Fisher Scientific) at 37 °C for 30 min with gentle agitation. The digested tissue was gently triturated using fire‐polished glass pipettes, passed through a 70 µm cell strainer to remove debris, and centrifuged at 300 × g for 5 min at 4 °C. The resulting single‐cell suspension was resuspended in 0.04% BSA/PBS for downstream processing. Single‐cell suspensions were prepared by enzymatic digestion of the tissue fragments in a digestion buffer. For each group, spinal cord tissues from three individual animals were pooled prior to tissue dissociation to ensure sufficient cell yield and to capture representative transcriptomic diversity while minimizing inter‐individual variability. Cell viability was assessed using trypan blue exclusion with a minimum viability of ≥85%. The cell concentration was adjusted to 700–1200 cells µL^−1^. Gel beads containing barcodes were combined with the cell suspension and enzyme mixture, and the mixture was encapsulated in oil‐based surfactant droplets within a microfluidic system, forming gel bead‐in‐emulsion (GEM) droplets. GEMs were collected, and the gel beads were dissolved to release the barcode sequences. Reverse transcription was performed to generate complementary DNA (cDNA) fragments, which were then tagged. Following bead fragmentation and oil droplet disruption, cDNA was used as a template for PCR amplification. PCR products from all GEMs were pooled to construct a standard sequencing library. Sequencing was conducted on the Illumina NovaSeq 6000 system at LC‐Bio Technology Co., Ltd (Hangzhou, China). Sequencing data are available in NCBI SRA (PRJNA1218622).

### Sequencing Data Quality Control and Quantification

Sequencing data were demultiplexed and converted into FASTQ format using Illumina bcl2fastq software (v2.20). Raw sequencing reads were aligned to the reference genome (specify version) using Cell Ranger (v3.1.0). This process included filtering out background cells and counting Unique Molecular Identifiers (UMIs) to quantify cell transcripts. The resulting data were organized into a gene‐barcode matrix to facilitate subsequent cell clustering and gene expression analysis. The cellranger aggr function was employed in order for integration and normalization of multi‐sample data. This function harmonized UMI counts across the samples, ensuring uniformity in data representation and mitigating batch effects.

### Cell Clustering and Annotation

Single‐cell RNA sequencing (scRNA‐seq) data were processed using Seurat (v4.0). Data normalization was performed with the NormalizeData function, and highly variable genes were identified using FindVariableFeatures. Principal Component Analysis (PCA) was carried out using RunPCA on these variable genes to capture principal components. Dimensionality reduction and clustering involved constructing a shared nearest neighbor (SNN) graph based on PCA results with the FindNeighbors function. Clustering was then executed with the Louvain algorithm using FindClusters. Uniform Manifold Approximation and Projection (UMAP) was employed for visualization with the RunUMAP function. Cell type classification was achieved using the SingleR package, comparing the log‐normalized data with reference cell type profiles. Annotations were manually adjusted based on known marker genes to enhance accuracy. Meantime, Fibroblast cells were further reclustered into subpopulations for in‐depth analysis.

### Visualization of Specific Genes on UMAP

The expression levels of specific genes of interest were extracted from normalized expression matrix, which were mapped onto the UMAP embeddings to illustrate gene‐specific expression patterns within the cellular landscape. The FeaturePlot function from Seurat was used to generate UMAP plots, where the expression of selected genes was overlaid on the UMAP representation. Expression values were color‐coded, with the color intensity reflecting gene expression level across cells.

### Scoring Custom Gene Sets

Custom gene sets were established according to specific biological contexts. scRNA‐seq data were preprocessed and normalized using Seurat (v4.0). Gene set scores were computed for each cell by averaging the expression levels of the constituent genes through the AddModuleScore function, which normalizes scores against a background distribution. To visualize gene set scores, boxplots were generated. Scores for each gene set were extracted and plotted with the ggplot2 package. Boxplots depicted the distribution, including range, median, and quartiles of gene set scores across various cell clusters or experimental conditions, facilitating comparative analysis of gene set activity.

### Gene Ontology (GO) Enrichment Analysis

GO enrichment analysis was performed using the R package ClusterProfiler (v4.0). Differentially expressed genes (DEGs) were identified and analyzed for GO term enrichment using the enrichGO function. This function maps gene identifiers to GO terms and evaluates the over‐representation of these terms.

### Dynamic RNA Velocity Analysis

After preprocessing and clustering fibroblast subpopulations with Seurat (v4.0), RNA velocity was estimated using scVelo (v0.2.4).^[^ ^18^ ^]^ Spliced and unspliced mRNA counts were used to calculate gene expression dynamics. The dynamic model in scVelo was applied to infer future expression states, and the results were visualized on UMAP.

### Pathway Enrichment Analysis

KEGG pathway enrichment was performed using the R package ClusterProfiler. DEGs were analyzed with the enrich KEGG function to identify enriched pathways. The Wilcoxon rank‐sum test was utilized to identify DEGs between cell subpopulations. Genes with a fold change >2 and adjusted *p*‐value <0.05 were considered as significance. Significant pathways were visualized with dot plots based on adjusted *p*‐values.

### Isolation of Rat Meningeal Fibroblasts

Meningeal fibroblasts were isolated from the spinal dura mater of adult Sprague‐Dawley rats. After euthanasia, the spinal column was exposed, and dura mater tissue was carefully dissected from the spinal cord. The tissue was placed in cold PBS, minced into 1–2 mm^3^ fragments, and digested with 0.1% collagenase I (ST2294, Beyotime, China) and 0.25% trypsin (C0201, Beyotime, China) at 37 °C for 30 min with gentle agitation. The digested suspension was filtered through a 70 µm strainer, centrifuged at 300 g for 5 min, and resuspended in Dulbecco's Modified Eagle Medium (DMEM, Thermo Fisher Scientific, USA) supplemented with 10% fetal bovine serum (FBS, Gibco, USA) and 1% penicillin‐streptomycin (SIGMA, USA). The cell suspension was subjected to a fibroblast‐specific purification step using a differential adhesion method. Cells were plated in culture dishes and incubated at 37 °C with 5% CO_2_ for 2 h. Non‐adherent cells were gently washed away, while fibroblasts that adhered to the culture dish were maintained in culture. Fibroblast colonies were observed within 3‐5 days.

### Cell Investigation In Vitro

Matrigel (356 237, CORNING, China) was diluted to an appropriate concentration and evenly distributed onto non‐adherent cell culture slides using a pipette, ensuring complete coverage of the surface. The coated slides were then incubated at 37 °C with 5% CO_2_ for 24 h to allow Matrigel to gel. Subsequently, a suspension of meningeal fibroblasts was prepared in DMEM supplemented with 10% FBS and 1% penicillin‐streptomycin. The cells were cultured to logarithmic growth phase, then harvested, resuspended in the prepared medium, and then was uniformly added to the gelled Matrigel‐covered slides. The slides were incubated at 37 °C with 5% CO_2_ for 24 h to allow cell attachment. Following incubation, the slides were carefully removed using sterile forceps. One half of the cell‐covered Matrigel was scraped off using a cell scraper along the centerline of the slide, marking the boundaries of the scraped area. HADA/HRR hydrogels of different mechanical strength were then prepared and injected into the scraped regions using a pipette, ensuring that the thickness of the hydrogels matched that of the Matrigel and that the boundaries were well‐aligned. After that, the medium was replaced to maintain the cell growth environment. The treated slides were returned to the incubator and cultured for another 48 h. Subsequently, relevant cell metrics were assessed and analyzed.

### 5‐Ethynyl‐2′‐Deoxyuridine (EdU) Immunofluorescence

To label newly synthesized DNA, cells were incubated with 10 µm EdU, prepared by diluting EdU stock solution (C10310‐3, Ribobio, China) in culture medium according to the manufacturer′s instructions. The EdU‐containing medium was added to the culture dish, and cells were then incubated at 37 °C with 5% CO_2_ for 48 h. After that, cells were washed twice with PBS, 5 min each time. Cells were fixed with 4% PFA for 20 min and subsequently washed with PBS to remove excess fixative. Permeabilization was performed by incubating the cells in 100 µL of 0.5% Triton X‐100 in PBS for 10 min on a shaker, followed by a single PBS wash for 5 min. Next, 100 µL of 1× Apollo 488 staining solution was added to each well, and the cells were incubated in the dark at 25 °C for 30 min on a shaker, followed by washing 3 times with 100 µL of 0.5% Triton X‐100 in PBS, 10 min each. 1 × Hoechst 33 342 solution, prepared by diluting reagent F 100:1 in deionized water, was added (100 µL per well) and incubated in the dark at 25 °C for 30 min. After incubation, cells were washed 1–3 times with 100 µL of PBS per well. Fluorescent images were acquired using an Axio Imager Z2 microscope (Zeiss, Germany) and a Leica STELLARIS STED confocal microscope (Leica Microsystems, Germany) for high‐resolution imaging.

### Flow Cytometry

For EdU flow cytometry, the medium was replaced with 10 µm EdU (Click‐iT EdU Pacific Blue Flow Cytometry Assay Kit, C10418, Thermo Fisher Scientific, USA), and the cells were incubated at 37 °C with 5% CO_2_ for 48 h after hydrogel injection in the in vitro. Cells were digested with 0.1% collagenase I and 0.25% trypsin at 37 °C for 10 min, followed sequentially by filtering through a 70 µm cell strainer, centrifuging at 1000 × *g* for 3 min, and resuspending in PBS. Cells were fixed with 4% PFA at 25 °C for 20 min, then permeabilized with 0.5% Triton X‐100 in PBS at 25 °C for 15 min after washing twice with PBS. Click‐iT reaction mixture was added according to the kit instructions and cells were resuspended in FACS buffer (PBS with 10% FBS) after cultivation in the dark for 30 min. EdU‐labeled cells were analyzed using flow cytometry after washing twice with Click‐iT wash buffer. The Pacific Blue dye was excited with a 405 nm laser and emission was detected at 450 nm. Data acquisition was performed using a BD FACSAria III flow cytometer (Becton & Dickinson, USA), and analysis was conducted using FlowJo software (v X.0.7). Appropriate gating and compensation with controls were used for accurate data analysis.

To assess the proportion of Itga11^+^/Il11ra1^+^ fibroblasts in vitro, cells on the hydrogel‐facing side were digested with 0.1% collagenase I and 0.25% trypsin at 37 °C for 10 min. The cell suspension was filtered through a 70 µm cell strainer and centrifuged at 1000 × *g* for 3 min. Cells were fixed with 4% PFA at 25 °C for 15 min, and were incubated for 30 min to block non‐specific fragment crystallizable receptor binding after resuspending in FACS buffer (PBS containing 10% FBS). After that, cells were washed twice with PBS and stained with primary antibodies: anti‐Itga11 (rabbit, 1:1000, Bioss) and anti‐Il11ra1 (mouse, 1:1000, KEMOBio) at 4 °C for 1 h. After primary antibody incubation, cells were washed twice with FACS buffer and then incubated with fluorophore‐conjugated secondary antibodies: anti‐rabbit IgG Alexa Fluor DAPI (1:500, Abcam) and anti‐mouse IgG Alexa Fluor APC (1:500, Abcam) at 4 °C in the dark for 30 min. After washing twice with FACS buffer, cells were resuspended in 500 µL buffer for flow cytometry analysis. Unstained and single‐stained controls were included to ensure accurate gating and exclude non‐specific staining. Data were acquired using a BD FACSAria III flow cytometer and analyzed with FlowJo software (vX.0.7). The proportion of double‐positive cells was determined relative to the total cell population.

### Western Blot

Regions containing hydrogel and cells in vitro were scraped with a cell scraper. The resulting material was digested at 37 °C for 30 min using a solution of 0.1% collagenase I and 0.25% trypsin at 37 °C for 10 min. The suspension was filtered through a 70 µm cell strainer, centrifuged at 1000 × *g* for 3 min. For in vivo samples, animals were deeply anesthetized and euthanized prior to perfusion with ice‐cold 0.9% saline. Fresh spinal cord tissue, ≈5 mm in length, was collected from the injury sites and rinsed with ice‐cold PBS.

Samples were lysed on ice using RIPA buffer (P0013B, Beyotime, China) supplemented with 1% Phenylmethylsulfonyl fluoride (PMSF, ST505, Beyotime, China), protease inhibitor cocktails (P1006, Beyotime, China), phosphatase inhibitor cocktails (P1046, Beyotime, China), and Ethylenediaminetetraacetic acid (EDTA, R0225, Beyotime, China). After lysis, the lysates were centrifuged at 4 °C at 12000 × *g* for 20 min, and the supernatants were collected for protein quantification according to the bicinchoninic acid (BCA) Protein Assay Kit (2 161 296, Thermo Fisher Scientific, USA).

Proteins were separated by sodium dodecyl sulfate‐polyacrylamide gel electrophoresis (SDS‐PAGE) and transferred to 0.22 µm polyvinylidene fluoride (PVDF) membranes (Millipore, USA). Membranes were blocked with 5% non‐fat milk in Tris‐buffered saline with Tween 20 (TBST, ST673, Beyotime, China) at 25 °C for 2 h. They were then incubated overnight at 4 °C with the primary antibodies (Table S2, Supporting Information). Following incubation, membranes were washed three times with TBST, with each wash lasting 10 min. The membranes were then incubated with HRP‐conjugated secondary antibodies for 2 h at 25 °C, followed by three additional washes with TBST. Protein bands were detected using the Tanon‐5200CE imaging system and enhanced chemiluminescence (ECL) detection reagents (1 863 095, Thermo Fisher Scientific, USA). Band intensities were quantified with ImageJ software (v1.53). Relative protein levels were normalized to GAPDH, and each experiment was conducted in triplicate (n = 3 per group).

### Cell Adhesion Force Measurement

Cell adhesion force measurement was conducted using a Dimension FastScan AFM (Bruker, Germany). To enable adhesion of fibroblasts to the cantilever beam, the cantilever was modified using the following protocol. Initially, the cantilever was subjected to plasma cleaning (PDC‐32G, Harrick Plasma, USA), followed by overnight incubation at 4 °C with Type I collagen (160 µg mL^−1^, PBS, Sigma, USA) to ensure coating of the upper end of the cantilever with these substances. AFM measurement was performed at 37 °C to preserve the cells' physiological environment. A non‐tapered silicon cantilever with a thickness of 600 nm (MLCT‐O10, Bruker, Germany) was employed. Prior to measurement, the precise spring constant of each cantilever was determined using the split‐slope method. Fibroblasts were prepared in a 24‐well plate with coverslips, which were then adhered to glass slides using adhesive to ensure stability. A sufficient volume of PBS was added to the coverslip to maintain a moist and stable environment, and the setup was placed on the AFM stage. Fibroblasts were optically monitored to ensure that only round, non‐spread cells were included in the adhesion measurements. Cells that spread prior to measurement were discarded. The cantilever and related parameters were adjusted to enable contact with fibroblasts. After ensuring stable contact, the cantilever was retracted from the surface to collect force‐distance curves.

### Bulk RNA‐seq

Spinal cord tissues (5 mm) were harvested from the injury sites of deeply anesthetized, perfused rats, rinsed with ice‐cold PBS, and washed three times with ice‐cold PBS. Total RNA was extracted using TRIzol (15 596 018, Thermo Fisher Scientific, USA). RNA concentration and purity were measured using a NanoDrop ND‐1000 spectrophotometer (NanoDrop, Wilmington, USA), and the integrity was assessed on a Bioanalyzer 2100 (Agilent, USA). Samples of concentrations >50 ng µL^−1^, RIN >7.0, and total RNA >1 µg were used for further analysis. Poly(A)^+^ mRNA was enriched via two rounds of Dynabeads Oligo(dT) purification (25‐61005, Thermo Fisher Scientific, USA). Fragmentation was performed at 94 °C for 5–7 min using the NEBNext Magnesium RNA Fragmentation Module (E6150S, NEB, USA). First‐strand cDNA synthesis was performed using SuperScript II (1 896 649, Thermo Fisher Scientific, USA), followed by second‐strand synthesis with E. coli DNA polymerase I (M0209, NEB, USA), RNase H (M0297, NEB, USA), and incorporation of dUTP (R0133, Thermo Fisher Scientific, USA). Double‐stranded DNA was end‐repaired, A‐tailed, adapter‐ligated, and size‐selected with magnetic beads. UDG (M0280, NEB, USA) was used for digestion, followed by PCR amplification: 95 °C for 3 min, 8 cycles of 98 °C for 15 s, 60 °C for 15 s, and 72 °C for 30 s, with a final extension at 72 °C for 5 min, yielding libraries of 300 ± 50 bp. Paired‐end sequencing (PE150) was performed on the Illumina Novaseq 6000 platform (LC‐Bio Technology, Hangzhou, China). Low‐quality bases were trimmed using Cutadapt (v.1.9), and reads were aligned with HISAT2 (v.2.2.1). Transcripts were assembled with StringTie (v.2.1.6) and merged with gffcompare (v.0.9.8). Transcript abundance (FPKM) was calculated using StringTie. DESeq2 (v.1.40.2) was used for differential expression analysis and results were visualized in volcano plots. GO enrichment was performed with clusterProfiler (v.4.0) and org.Hs.eg.db (v.3.13.0). A Venn diagram was generated using the VennDiagram R package (v.1.6.20) to visualize the overlap of DEGs between groups. The heatmap was generated using the pheatmap R package (v.1.0.12). Z‐scores of gene expression was calculated and clustered to visualize the expression patterns across samples.

### Assessment of MMP7 Function

To evaluate the effects of MMP7 protein, experiments were conducted both in vitro and in vivo. For in vitro experiments, MMP7 protein (100 µM, HY‐P700574, MedChem‐Express, USA) or its small molecule inhibitor MMP‐7‐IN‐1 (1 µM, HY‐151540, MedChem‐Express, USA) was added to the culture medium. DMSO was used as the solvent to prepare stock solutions at concentrations of 100 or 10 mm. Quantified stock solutions were added to the culture medium as needed, with an equal volume of DMSO used for control samples. For in vivo experiments, MMP7 protein (10 mg kg^−1^) or MMP‐7‐IN‐1 (1 mg/kg) was directly added in HADA/HRR hydrogels before injection into the injury site. Control rats received an equal volume of DMSO. Post‐injection, the rats were monitored and recorded according to standard procedures to ensure reliable data collection.

### Assessment of β‐Catenin Phosphorylation

To assess the role of β‐Catenin phosphorylation, fibroblasts seeded onto hydrogels were treated with β‐Catenin phosphorylation inhibitor Laduviglusib (10 µM, HY‐10182, MedChem‐Express, USA) or β‐Catenin phosphorylation enhancer (E)‐Ferulic acid (50 µm, HY‐N0060B, MedChem‐Express, USA) in the medium. DMSO was used as a solvent to prepare stock solutions of the inhibitors at 100 mm. Appropriate aliquots of these stock solutions were added to the culture medium, while control samples received an equivalent volume of DMSO, ensuring accurate reagent concentrations and maintained consistency across experimental conditions.

### Assessment of LRP6 Phosphorylation

For the in vitro studies, fibroblasts seeded onto HADA/HRR hydrogels were treated with LRP6 phosphorylation enhancer HLY78 (20 µm, HY‐122816, MedChem‐Express, USA) or LRP6 phosphorylation inhibitor Salinomycin (1 µm, HY‐15597, MedChem‐Express, USA) in the medium. DMSO was used as a solvent for preparing stock solutions of the drugs at 10 mm. Appropriate aliquots of these stock solutions were added to the culture medium, while control samples received an equivalent volume of DMSO, ensuring accurate drug concentrations and consistency across experimental conditions. For the in vivo studies, rats were randomly assigned to HADA/HRR hydrogel groups and SCI group, with 5 rats in each group. At the start of the experiment, rats received intrathecal injections of HLY78 (10 mg/kg) or Salinomycin (10 mg kg^−1^) at the site of spinal cord injury. Control rats received an equivalent volume of DMSO.

### Anterograde Tracing with AAV9 Virus

To investigate axonal regeneration of propriospinal neurons after SCI, anterograde tracing using AAV9 virus was performed at 0 dpi and 2 wpi. A midline incision was made along the dorsal surface to expose the T4‐T6 spinal cord segments. Injections were performed at eight specific sites, with mediolateral coordinates of ±0, ±0.4, ±0.8, and ±1.2 mm from the spinal cord midline, and a dorsoventral depth of 0.8–1.2 mm. At each site, 0.5 µL of AAV9 virus was delivered using a microinjection pump at a rate of 40 nL min^−1^. To prevent backflow, the needle was left in place for 10 min after each injection, carefully avoiding blood vessels. Postoperatively, meloxicam was administered for analgesia. Two weeks after the injection, rats were perfused, and tissues were collected for analysis. The sections were selected in a systematic manner (every 15th section) to minimize bias. Fluorescence images were acquired using a Zeiss fluorescence microscope to evaluate the extent of axonal regeneration in propriospinal neurons. For quantification, a total of three rats per group were analyzed. The fluorescence signal intensity of traced axons was normalized to the mean value obtained from sections rostral to the lesion site within the same animal, thereby reducing variability due to injection efficiency or viral spread.

### Evaluation of Neural Fiber Quality

To comprehensively evaluate axonal regeneration at 4 wpi, a combined analysis of neural fiber quality was conducted based on the number of AAV9‐labeled fibers and their average length. Specifically, the number of AAV9‐labeled fibers was plotted on the x‐axis and their average length on the y‐axis to establish an XY coordinate system. Within this system, the upper‐right quadrant was designated as the high‐quality (Hi‐Q) zone, representing fibers with both high abundance and extended length, whereas the lower‐left quadrant was defined as the low‐quality (Lo‐Q) zone, characterized by low fiber numbers and shorter lengths. Data from each experimental group were mapped onto this coordinate system to enable visual comparison of axonal regeneration across groups. Statistical analyses were subsequently performed, and the results were presented using scatter plots to illustrate the distribution of fiber numbers and lengths.

### Transmission Electron Microscopy

After deep anesthesia and euthanasia, rats underwent cardiac perfusion with a fixative solution containing equal parts of 2% glutaraldehyde and 4% PFA. A 2 mm segment of the spinal cord was then excised from the surgical site and submerged in the fixative solution at 4 °C for 1 h. After that, the tissues were exposed to 1% osmium tetroxide for another hour. To dehydrate the samples, a sequential ethanol series was employed before embedding in Epon resin. Ultra‐thin sections, with a thickness of 80 nm, were cut using a Reichert ultramicrotome (Reichert E, Co, Austria). These sections were stained appropriately and examined using a Philips CM 10 transmission electron microscope (Philips, The Netherlands).

## Conflict of Interest

The authors declare no conflict of interest.

## Supporting information



Supporting Information

## Data Availability

The data that support the findings of this study are available from the corresponding author upon reasonable request.
