# Supplementary material for: Targeting Fibrotic Scarring by Mechanoregulation of Il11ra1+/Itga11+ Fibroblast Patterning Promotes Axon Growth after Spinal Cord Injury
Source: Adv Sci (Weinh). 2025 Sep 9;12(44):e13476. doi: 10.1002/advs.202513476 (PMC12667452; doi:10.1002/advs.202513476)
Supplement: Supplementary file 1 — Supporting Information [file ADVS-12-e13476-s001.docx]

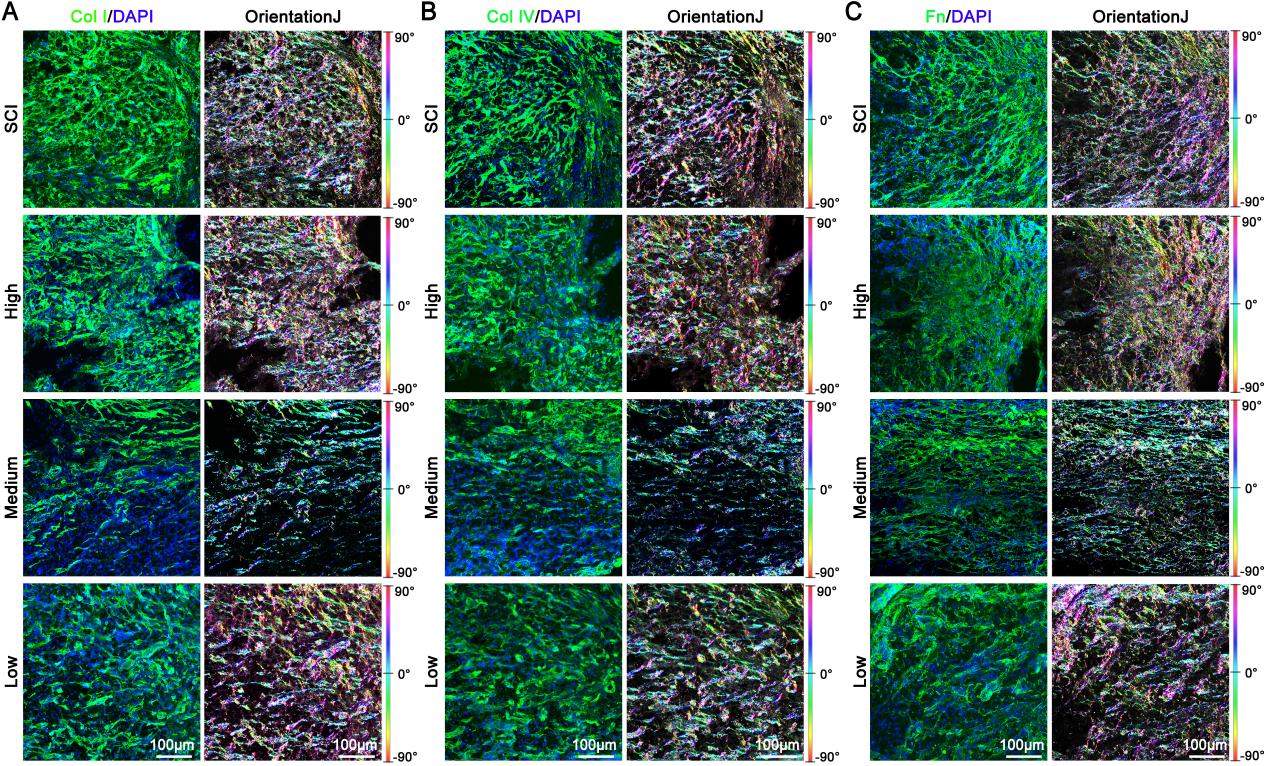


**Figure S1 (related to Figure 2). HADA/HRR Medium hydrogel promoted the aligned organization of ECM.**

(A-C) Representative immunostaining images (in the left side of each panel) and corresponding color-coded images (using OrientationJ and in the right side of each panel) of ECM [collagen Ⅰ (Col Ⅰ, A), Collagen Ⅳ (Col Ⅳ, B) and fibronectin (Fn, C)] in the injury boundary along the longitudinal sections of the spinal cord at 2 wpi. The orientations of each component of ECM were indicated by the color scale bars.


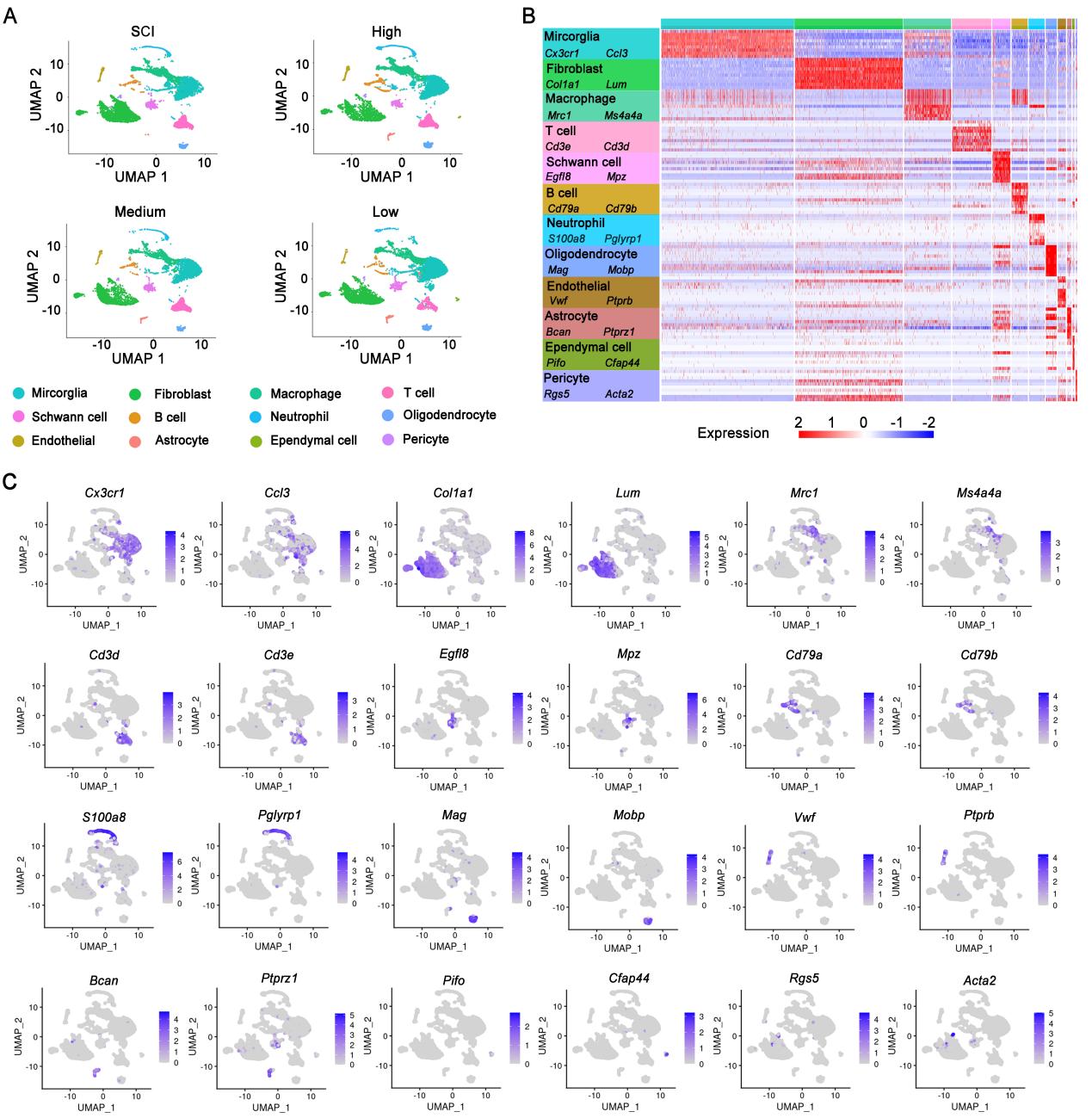


**Figure S2 (related to Figure 3). scRNA-seq identified multiple cell types.**

(A) UMAP plots showing cell components dissociated from the injured spinal cord tissues in different groups based on scRNA-seq. (B) Heatmap displaying the differentially expressed genes in each cell type. (C) UMAP plots revealing marker genes for each cell type.


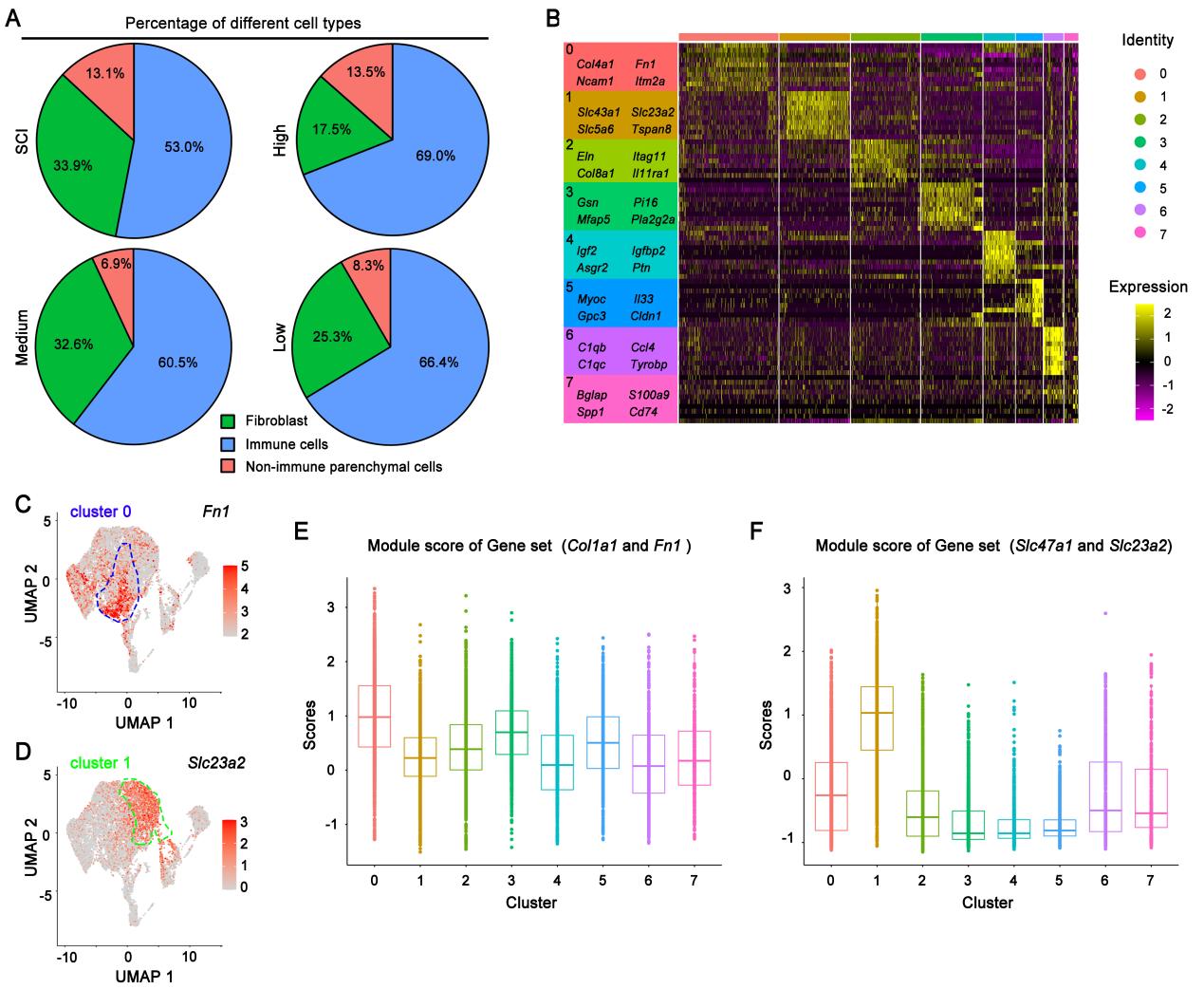


**Figure S3 (related to Figure 3). scRNA-seq identified multiple fibroblast subsets.**

(A) Pie charts showing cell components dissociated from the injured spinal cord tissue of different groups based on scRNA-seq. (B) Heatmap displaying the differentially expressed genes in each fibroblast cluster. (C) UMAP plots revealing the expression levels of *Fn1* in fibroblast clusters based on scRNA-seq. (D) UMAP plots revealing the expression levels of *Slc23a2* in fibroblast clusters based on scRNA-seq. (E) The module score showed the expression level of a specific gene module (based on *Col1a1* and *Fn1*) within fibroblast clusters. (F) The module score showed the expression level of a specific gene module (based on *Slc47a1* and *slc23a2*) within fibroblast clusters.


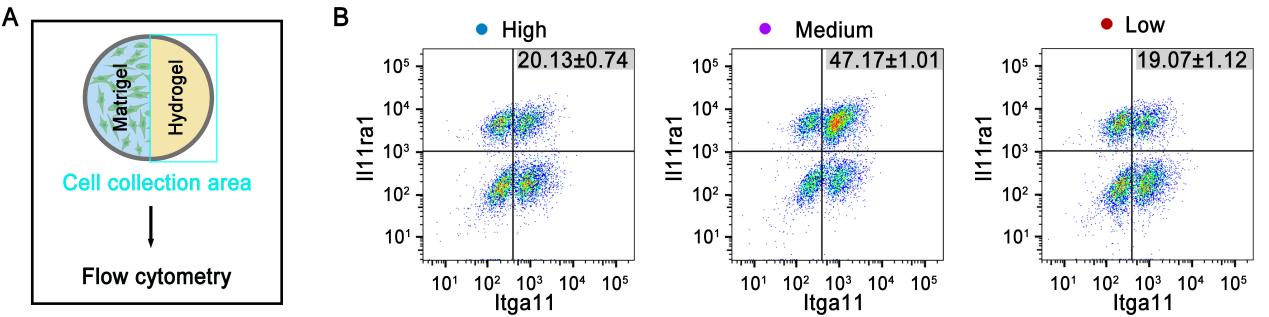


**Figure S4 (related to Figure 4). HADA/HRR Medium hydrogel induced the Itga11^+^/Il11ra1^+^ fibroblast subset *in vitro*.**

(A) Schematic of cell collection area for flow cytometry *in vitro*. (B) Flow cytometric analysis of the percentage of Itgall^+^ /Il11ra1^+^ fibroblasts migrated in the hydrogels with High, Medium and Low mechanical strength *in vitro*.


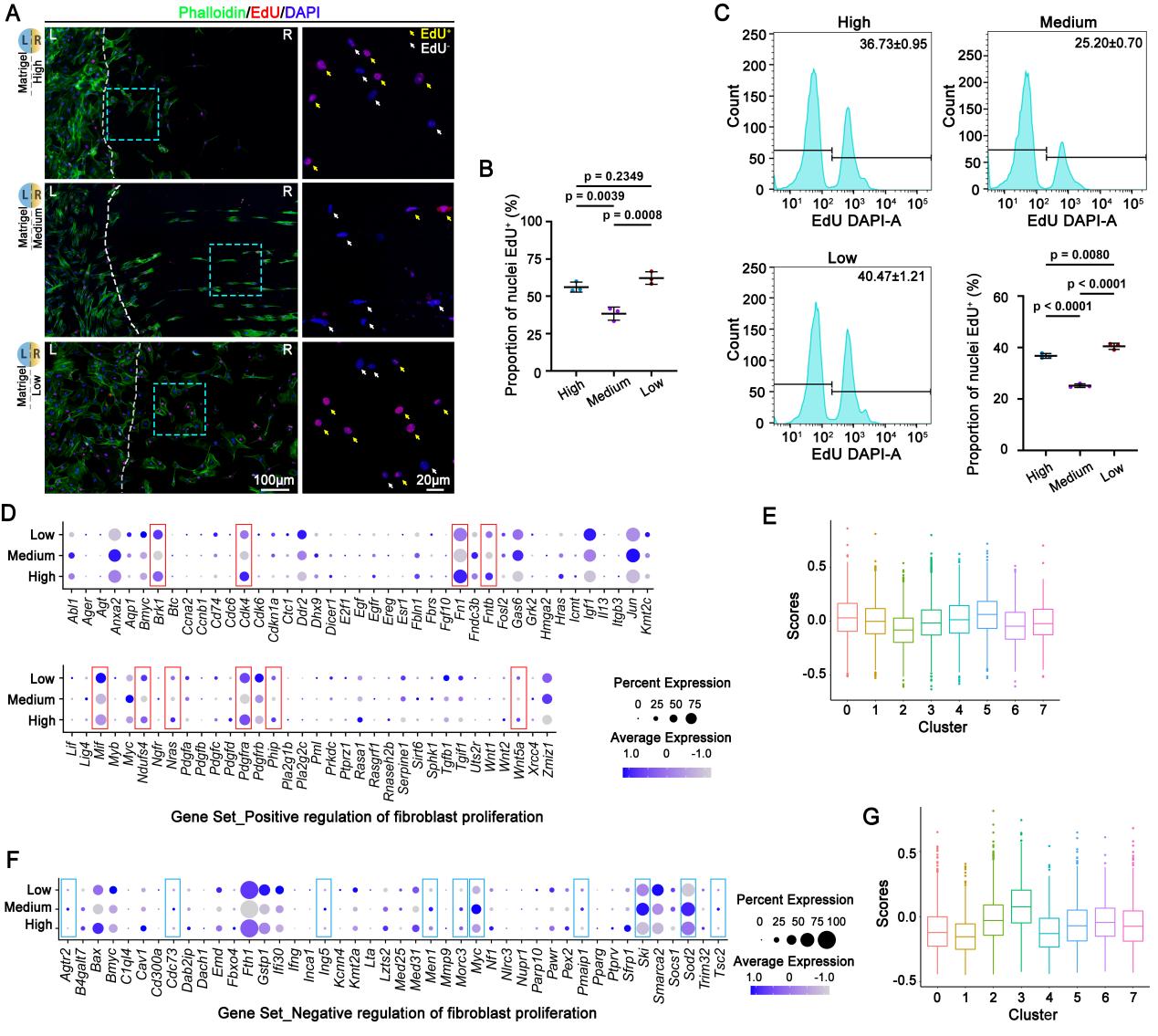


**Figure S5 (related to Figure 4). HADA/HRR Medium hydrogel reduced the proliferation ability of fibroblasts.**

(A) Representative images of the co-immunostaining of EdU and phalloidin in the fibroblasts migrating on the hydrogels with different mechanical strength. The blue boxed regions were enlarged views of EdU signal and DAPI. White dashed lines indicate the interface between the Matrigel and the hydrogel. L, left; R, right. (B)The quantification of the proportion of EdU^+^ nuclei. N = 3 samples. (C) Flow cytometric analysis of EdU^+^ fibroblasts migrating on the hydrogels with different mechanical strength *in vitro*. N = 3 samples. (D) Dot plot showing the gene expression of the 'positive regulation of fibroblast proliferation' gene set in spinal cord tissue from different groups based on scRNA-seq. The red boxes indicate genes that were downregulated in the HADA/HRR Medium hydrogel group. (E) The module score showing the expression level of a specific gene module (based on downregulated genes in the HADA/HRR Medium hydrogel group in D) within fibroblast clusters. (F) Dot plot showing the gene expression of the negative regulation of fibroblast proliferation' gene set in spinal cord tissue from different groups based on scRNA-seq. The red boxes indicated genes that were upregulated in the HADA/HRR Medium hydrogel group. (G) The module score showed the expression level of a specific gene module (based on based on upregulated genes in the HADA/HRR Medium hydrogel group in F) within fibroblast clusters. Data were shown as the mean ±SD; P values were determined by two-tailed one-way ANOVA with Tukey’s multiple-comparisons test.


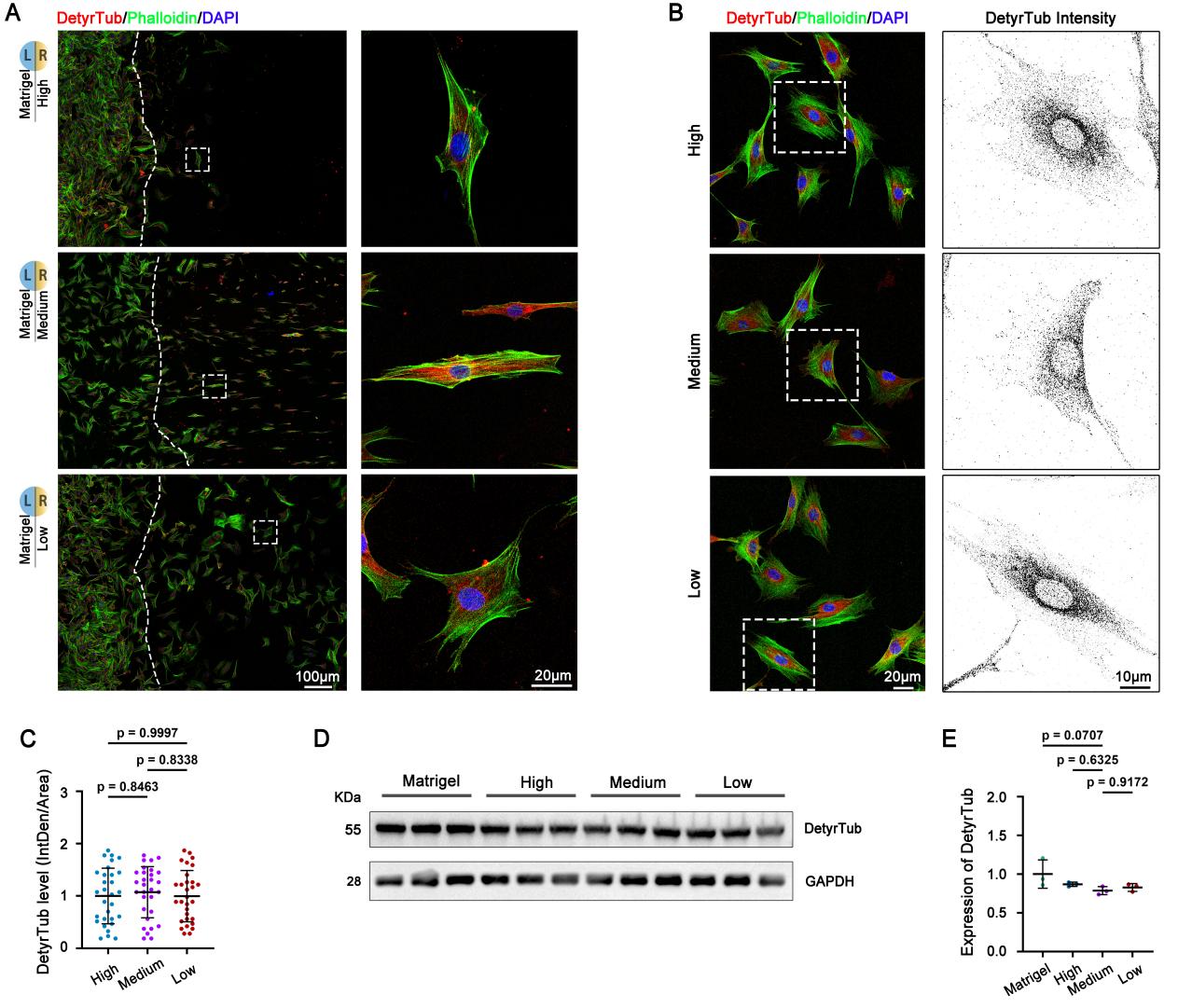


**Figure S6 (related to Figure 4). The mechanical strength gradient of HADA/HRR hydrogels manipulated fibroblast polarization.**

(A) Representative images of the co-immunostaining of DetyrTub and phalloidin in the fibroblasts migrating on the hydrogels with different mechanical strength. The White boxed regions were enlarged on the right. White dashed lines indicated the interface between the Matrigel and the hydrogel. The panel of the corresponding group in A was identical to Figure 4E. (B) Representative images the co-immunostaining of DetyrTub and phalloidin in the fibroblasts seeded directly on the hydrogels with different mechanical strength. The White boxed regions were enlarged in the grayscale to represent the intensity of DetyrTub. (C) The quantification of the relative fluorescence intensity of DetyrTub. N = 30 cells. (D-E) Representative chopped Western blot images (D) and quantitation (E) displaying the expression of DetyrTub in the fibroblasts seeded directly on the hydrogels. N = 3 samples. Data were shown as the mean ± SD; P values were determined by two-tailed one-way ANOVA with Tukey’s multiple-comparisons test.


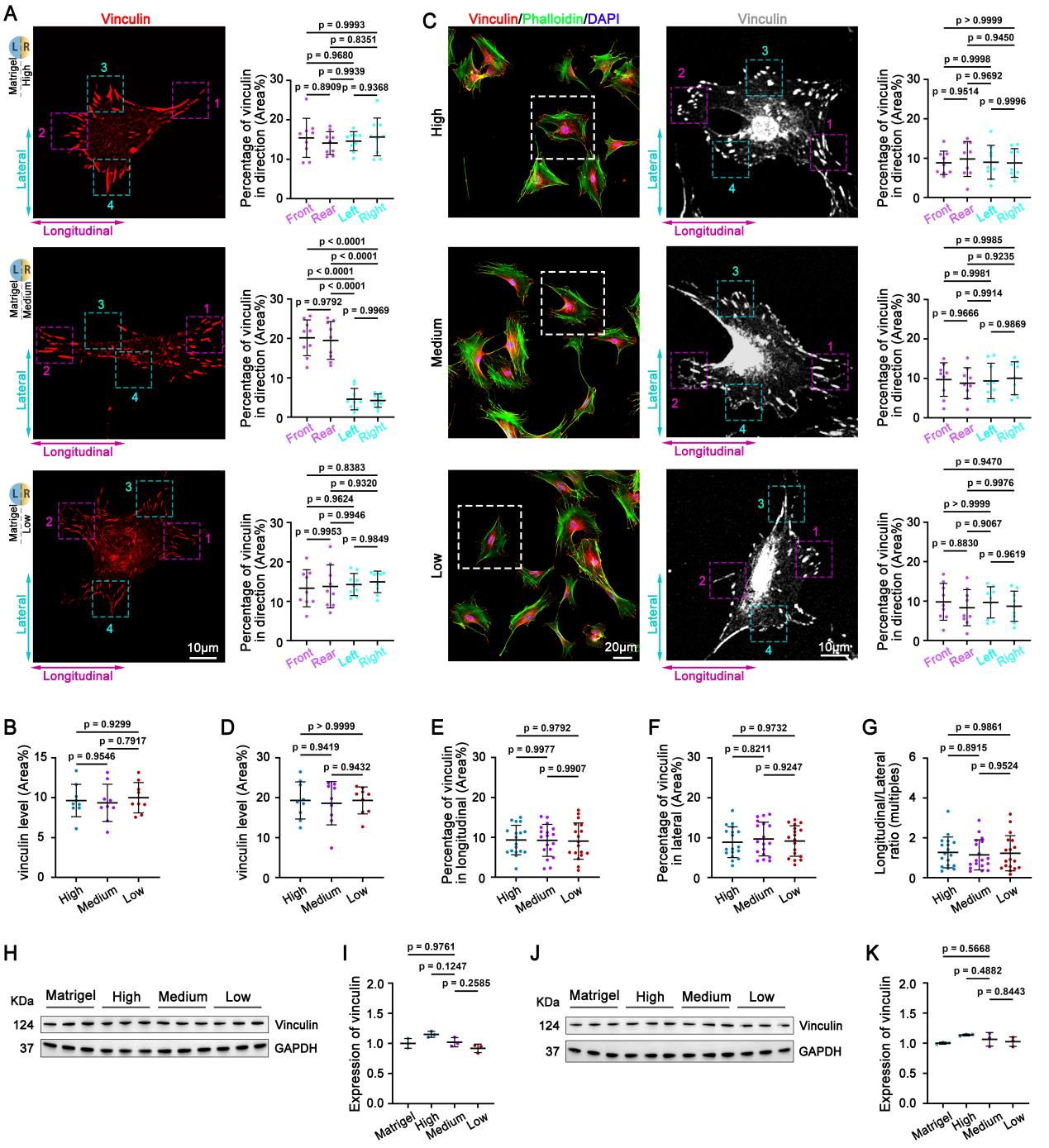


**Figure S7 (related to Figure 4). The mechanical strength gradient of HADA/HRR hydrogels manipulated the distribution of vinculin in fibroblasts.**

(A) Representative immunostaining images showing the expression of vinculin in fibroblasts migrating on the hydrogels (right side) with different mechanical strength. The purple boxes and numbers (1 and 2) indicate the expression of vinculin in the longitudinal direction, while the cyan boxes and numbers (3 and 4) indicate the expression of vinculin in the lateral direction. The quantifications of the relative fluorescence intensity of vinculin in different directions were listed on the right. N = 9 cells, respectively. (B) The quantitative analysis of the relative fluorescence intensity of vinculin in the fibroblasts migrating on the hydrogels with different mechanical strength. N = 9 cells. (C) Representative images of the co-immunostaining of vinculin and phalloidin in the fibroblasts seeded directly on hydrogels with different mechanical strength. The white boxes regions were the enlarged views of vinculin signal. The purple boxes and numbers (1 and 2) indicate the expression of vinculin in the longitudinal direction, while the cyan boxes and numbers (3 and 4) indicate the expression of vinculin in the lateral direction. The quantifications of the relative fluorescence intensity of vinculin in different directions were listed on the right. N = 9 cells, respectively. (D) The quantitative analysis of the relative fluorescence intensity of vinculin in the fibroblasts seeded directly on the hydrogels with different mechanical strength. N = 9 cells. (E-G) The graphs quantify the relative fluorescence intensity of vinculin in both the longitudinal (E) and lateral (F) directions, as well as the longitudinal/lateral ratio (G) of the fibroblasts seeded directly on the hydrogels with different mechanical strength. N = 9 cells, respectively. (H-I) Representative chopped Western blot images (H) and quantitation (I) revealing the expression of vinculin in the fibroblasts on the hydrogel side (the right side) with mechanical strength gradient, which migrated from the Matrigel side (the left side). N = 3 samples. (J-K) Representative chopped Western blot images (J) and quantitation (K) revealing the expression of vinculin in the fibroblasts seeded directly on hydrogels with different mechanical strength. N = 3 samples. Data were shown as the mean ± SD; P values were determined by two-tailed one-way ANOVA with Tukey’s multiple-comparisons test.


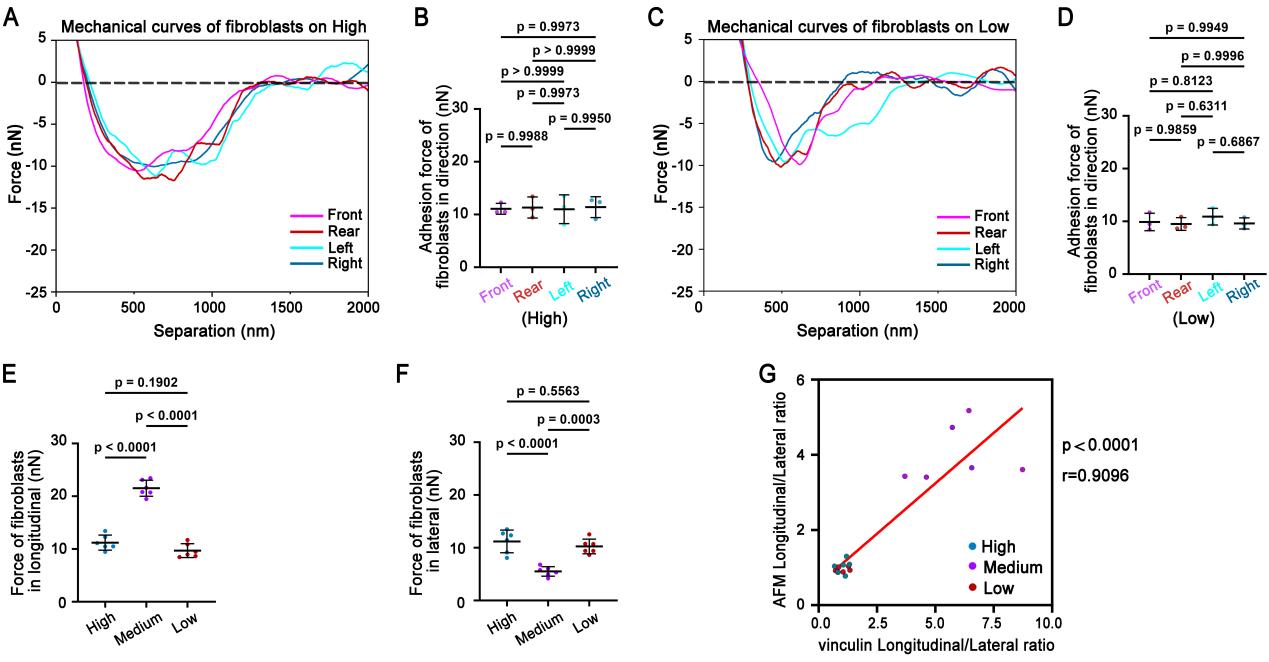


**Figure S8 (related to Figure 4). HADA/HRR Medium hydrogel generated adhesion forces along the direction of the migration.**

(A) Mechanical curves of fibroblasts on High hydrogel in different directions generated by AFM. (B) The quantitative analysis of the adhesion force of the fibroblast in various directions on High hydrogel. N = 3 samples. (C) Mechanical curves of fibroblasts on Low hydrogel in different directions generated by AFM. (D) The quantitative analysis of the adhesion force of the fibroblast in various directions on Low hydrogel. N = 3 samples. (E-F) The quantitative analysis of the adhesion force in both the longitudinal (E) and lateral (F) orientations of fibroblasts migrated on the hydrogels with different mechanical strength. N = 3 samples, respectively. (G) A linear regression model relating AFM longitudinal/lateral ratio to AFM longitudinal/lateral ratio. Data were shown as the mean ± SD; P values were determined by two-tailed one-way ANOVA with Tukey’s multiple-comparisons test.


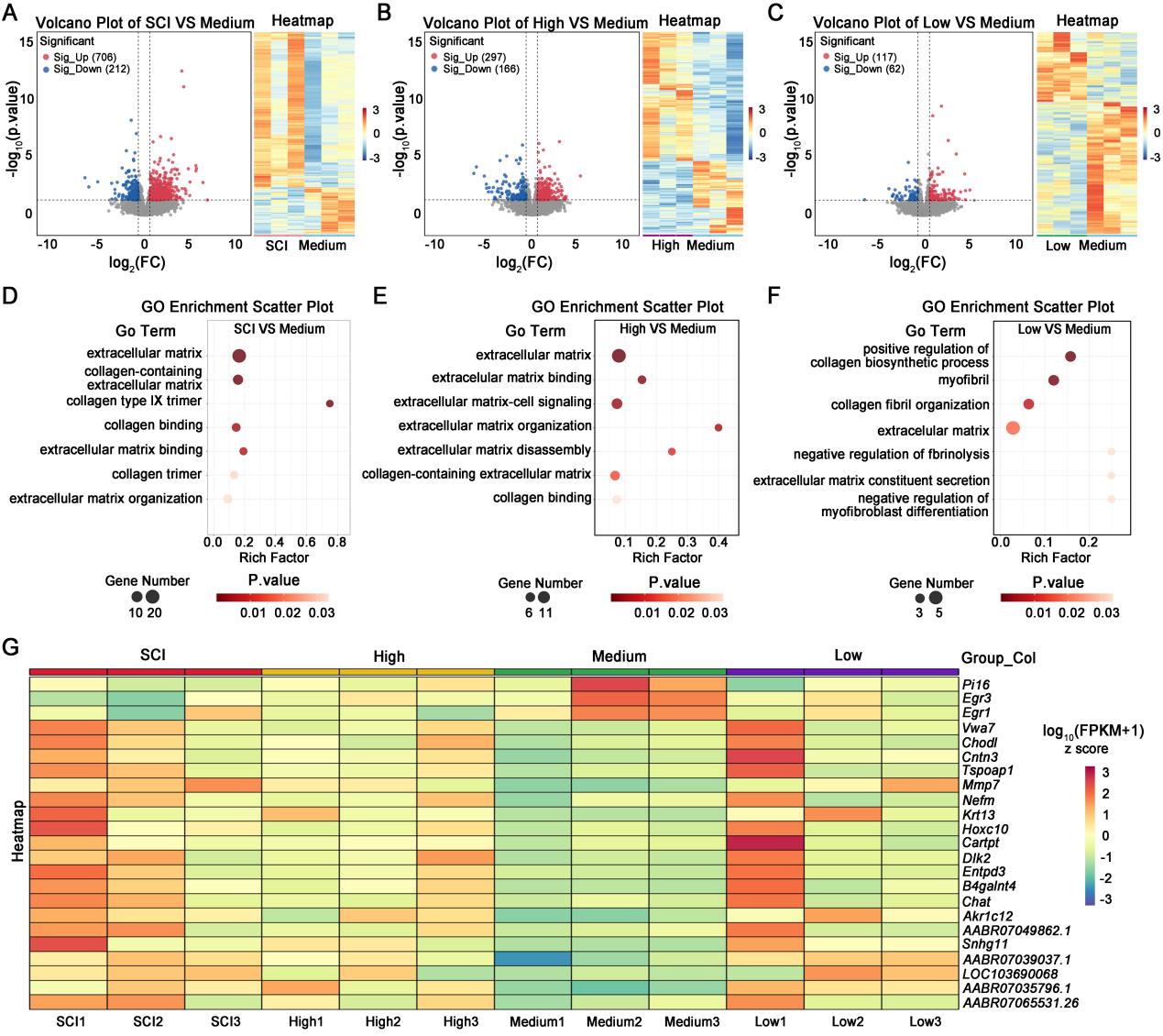


**Figure S9 (related to Figure 5). Bulk RNA-seq identified genes responsive to the mechanical strength gradient.**

(A-C) Volcano plots and heatmaps showing Differentially Expressed Genes (DEGs) between the SCI and Medium groups (A), High and Medium groups (B), and Low and Medium groups (C) from the bulk RNA-seq. (D-F) ECM-related Gene Ontology (GO) enrichment scatter plot of the DEGs between the SCI and Medium groups (D), High and Medium groups (E), and Low and Medium groups (F) from the bulk RNA-seq. (G) Heatmap showing the expression level of the 23 overlapped DEGs among groups.


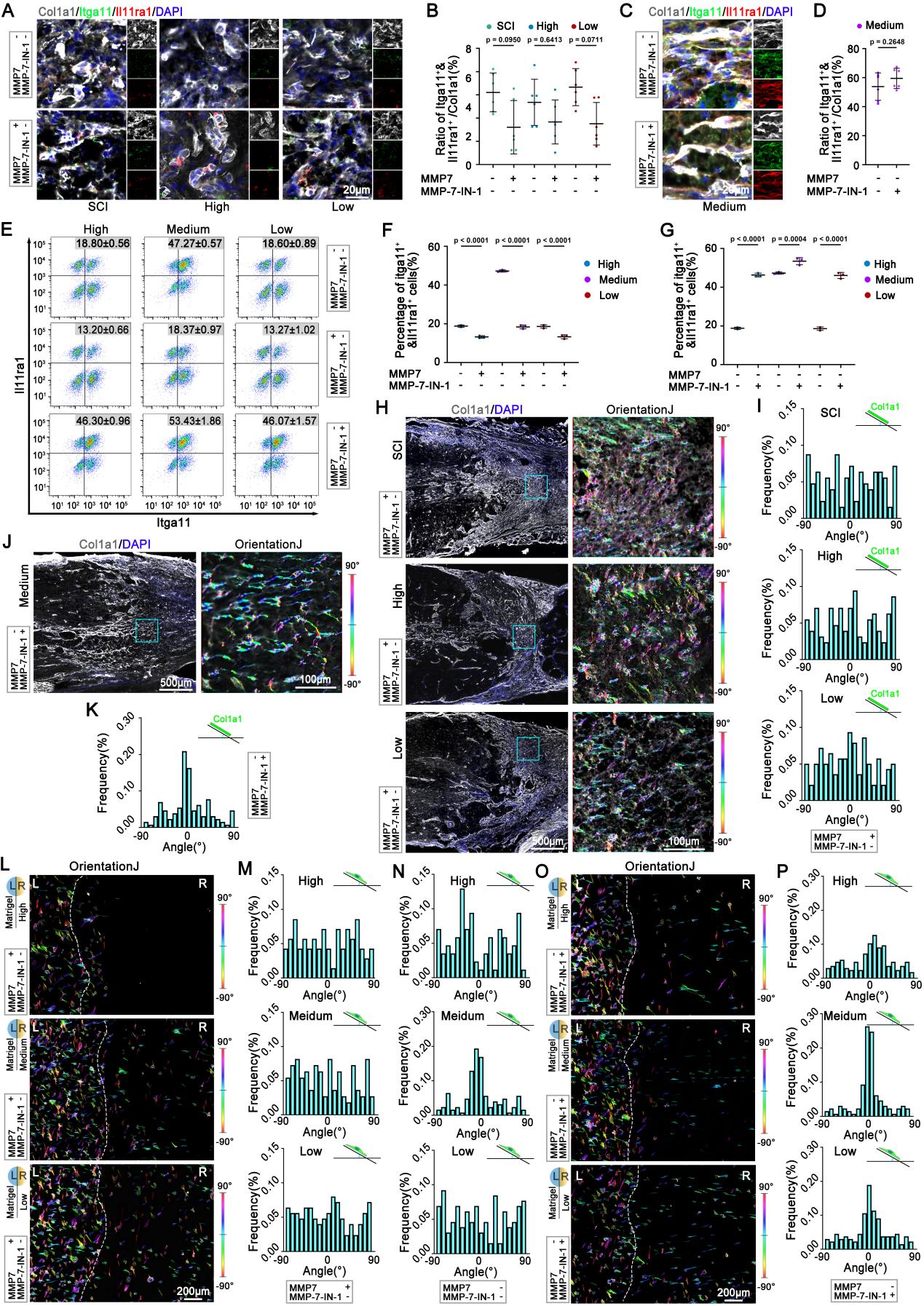


**Figure S10 (related to Figure 5). MMP7 as a key factor in regulating the specific behavior of fibroblasts.**

(A) Representative multichannel immunostaining images showing the expression of Col1a1, Itga11, and Il11ral in the longitudinal sections of the spinal cord at 2 wpi following the intervention with MMP7 on SCI, High hydrogel and Low hydrogel group, respectively. The separate channels were presented in the right rows. (B) The quantifications of the proportion of Itga11^+^/Il11ra1^+^ cells within the Col1a1^+^ population following the intervention with MMP7 on SCI, High hydrogel and Low hydrogel group. N = 6 animals. (C) Representative multichannel immunostaining images showing the expression of Col1a1, Itga11, and Il11ral in the longitudinal sections of the spinal cord sections at 2 wpi following the intervention with MMP-7-IN-1 on Medium hydrogel group. The separate channels were presented in the right rows. (D) The quantifications of the proportion of Itga11^+^/Il11ra1^+^ cells within the Col1a1^+^ population following the intervention with MMP-7-IN-1 on Medium hydrogel group. N = 6 animals. (E) Flow cytometric analysis of Itgall^+^ /Il11ra1^+^ fibroblasts *in vitro* following the intervention of MMP7 and MMP-7-IN-1, respectively. (F) The quantifications of flow cytometric analysis of Itgall^+^ Il11ra1^+^ fibroblasts *in vitro* following the intervention of MMP7. N = 3 samples. (G) The quantifications of flow cytometric analysis of Itgall^+^ Il11ra1^+^ fibroblasts *in vitro* following the intervention of MMP-7-IN-1. N = 3 samples. (H) Representative multichannel immunostaining images revealing the expression of Col1a1 in the longitudinal sections of the spinal cord at 2 wpi following the intervention with MMP7 on SCI, High hydrogel and Low hydrogel group, respectively. The blue boxed regions were the higher magnifications of the color-coded images revealing the orientation of Col1a1 using OrientationJ. The Color scale bars represent Col1a1 orientation. (I) The quantifications of the orientations of Col1a1 following the intervention with MMP7 on SCI, High hydrogel and Low hydrogel group, respectively. (J) Representative multichannel immunostaining images revealing the expression of Col1a1 in the longitudinal sections of the spinal cord at 2 wpi following the intervention with MMP-7-IN-1 on Medium hydrogel group. The blue boxed regions were the higher magnifications of the color-coded images revealing the orientation of Col1a1 using OrientationJ. The Color scale bar represents Col1a1 orientation. (K) The quantifications of the orientations of Col1a1 following the intervention with MMP-7-IN-1 on Medium hydrogel group. (L) Representative images displaying the migration and the distribution of fibroblasts following the intervention with MMP7 in different groups. The orientations of the fibroblasts were color-coded using OrientationJ, which were indicated by the color scale bars. White dashed lines indicated the interface between the Matrigel and the hydrogel. L, left; R, right. (M-N) The histogram represents the distribution of the orientations of the fibroblasts following the intervention with MMP7 in each group. (O) Representative images displaying the migration and the distribution of fibroblasts following the intervention with MMP-7-IN-1 in different groups. The orientations of the fibroblasts were color-coded using OrientationJ, which were indicated by the color scale bars. White dashed lines indicate the interface between the Matrigel and the hydrogel. L, left; R, right. (P) The histogram represents the distribution of the orientations of the fibroblasts following the intervention with MMP-7-IN-1 in each group. Data were shown as the mean ± SD; P values were determined by two-tailed paired t-tests.


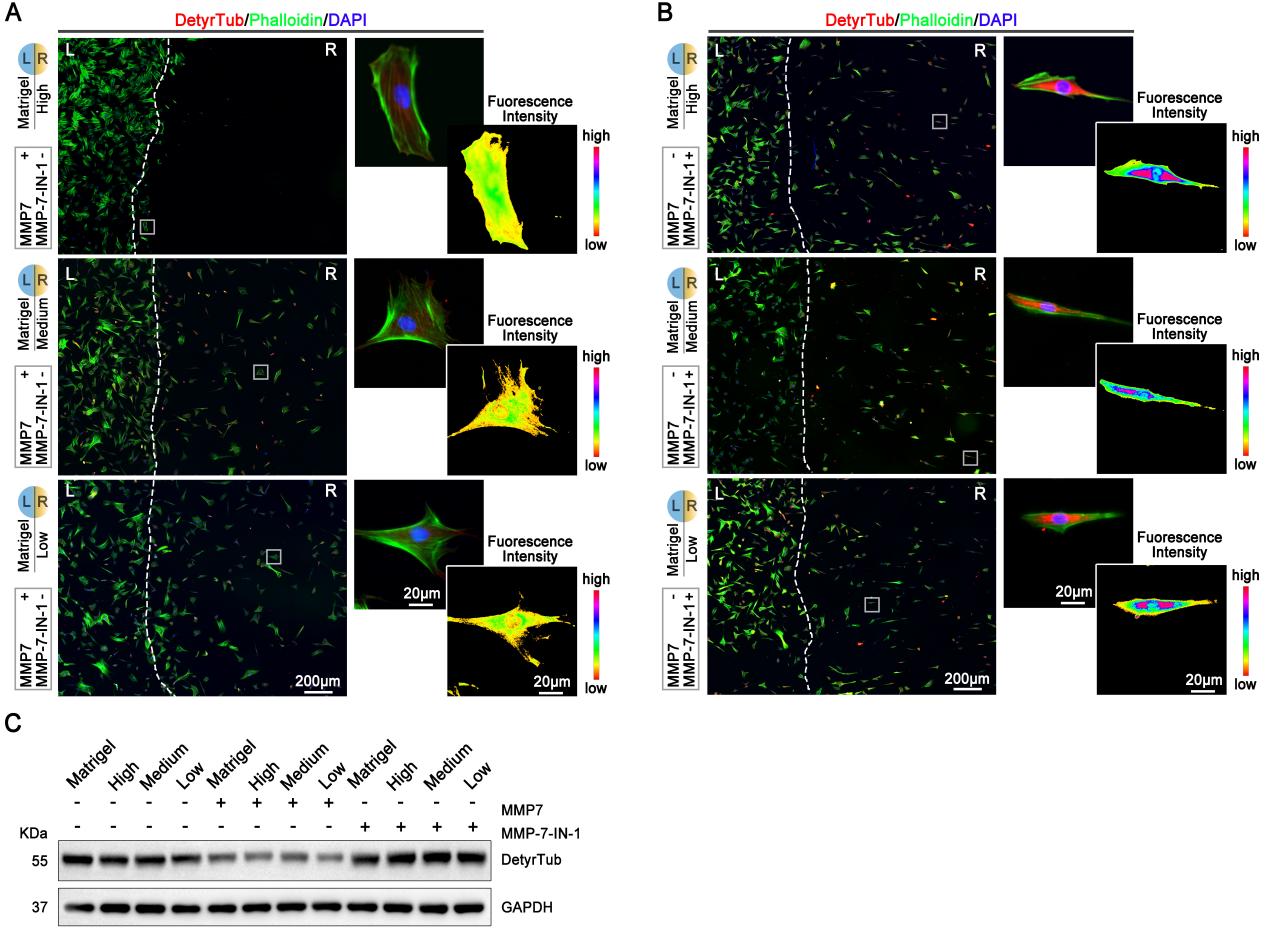


**Figure S11 (related to Figure 5). MMP7 as a key factor in regulating polarization of fibroblasts.**

(A) Representative images of the co-immunostaining of DetyrTub and phalloidin in the fibroblasts from different groups following the intervention with MMP7. The white boxes highlight the enlarged area, and the fluorescence intensity of DetyrTub was shown on the right. White dashed lines indicate the interface between the Matrigel and the hydrogel. L, left; R, right. (B) Representative images of the co-immunostaining of DetyrTub and phalloidin in the fibroblasts from different groups following the intervention with MMP-7-IN-1. The white boxes highlight the enlarged area, and the fluorescence intensity of DetyrTub was shown on the right. White dashed lines indicate the interface between the Matrigel and the hydrogel. L, left; R, right. (C) Representative chopped Western blot images showing the expression of DetyrTub *in vitro* following the intervention with MMP7 and MMP-7-IN-1 respectively.

**
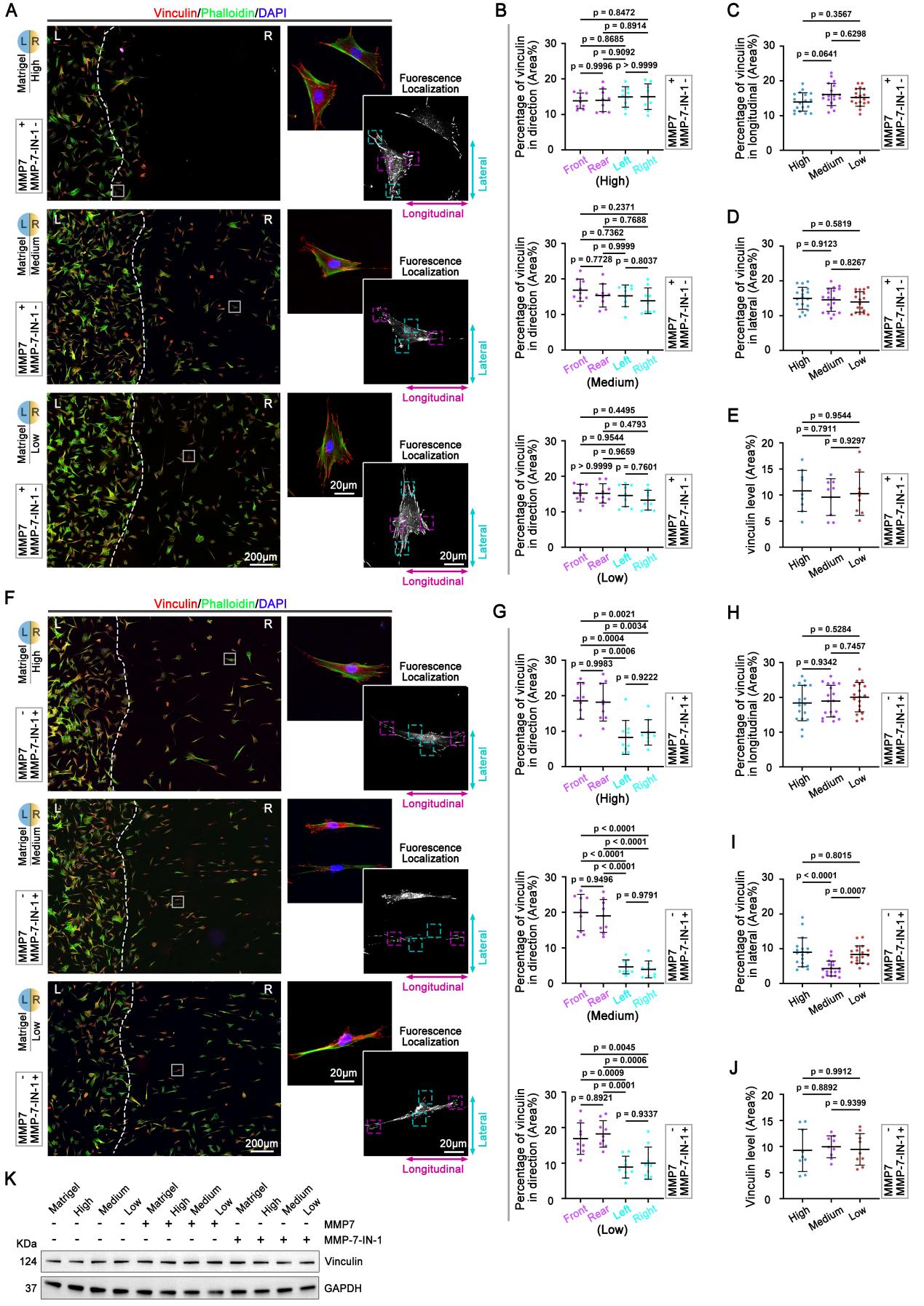
**

**Figure S12 (related to Figure 5). MMP7 is a key factor in regulating the distribution of vinculin of fibroblasts.**

(A) Representative images of the co-immunostaining of vinculin and phalloidin in the fibroblasts in different groups following intervention with MMP7. The white boxed regions were enlarged on the right. The signal of vinculin was shown on the grayscale for the intensity quantitation in the lateral (cyan boxes) and the longitudinal (purple boxes) directions. White dashed lines indicate the interface between the Matrigel and the hydrogel. L, left; R, right. (B) The quantifications of relative fluorescence intensity of vinculin in different direction in the fibroblasts in different groups following intervention with MMP7. N = 9 cells, respectively. (C-D) The quantification of the relative fluorescence intensity of vinculin in both the longitudinal (C) and lateral (D) orientations following intervention with MMP7. N = 9 cells, respectively. (E) The quantitative analysis of the relative fluorescence intensity of vinculin in different hydrogel groups following intervention with MMP7. N = 9 cells. (F) Representative images of the co-immunostaining of vinculin and phalloidin in the fibroblasts in different groups following intervention with MMP-7-IN-1. The white boxed regions were enlarged on the right. The signal of vinculin was shown on the grayscale for the intensity quantitation in the lateral (cyan boxes) and the longitudinal (purple boxes) directions. White dashed lines indicate the interface between the Matrigel and the hydrogel. L, left; R, right. (G) The quantifications of relative fluorescence intensity of vinculin in different direction in the fibroblasts in different groups following intervention with MMP-7-IN-1. N = 9 cells, respectively. (H-I) The quantification of the relative fluorescence intensity of vinculin in both the longitudinal (H) and lateral (I) orientations following intervention with MMP-7-IN-1. N = 9 cells, respectively. (J) The quantitative analysis of the relative fluorescence intensity of vinculin in different hydrogel groups following intervention with MMP-7-IN-1. N = 9 cells. (K) Representative chopped Western blot images showing the expression of vinculin *in vitro* following the intervention with MMP7 and MMP-7-IN-1 respectively. Data were shown as the mean ± SD; P values were determined by two-tailed one-way ANOVA with Tukey’s multiple-comparisons test


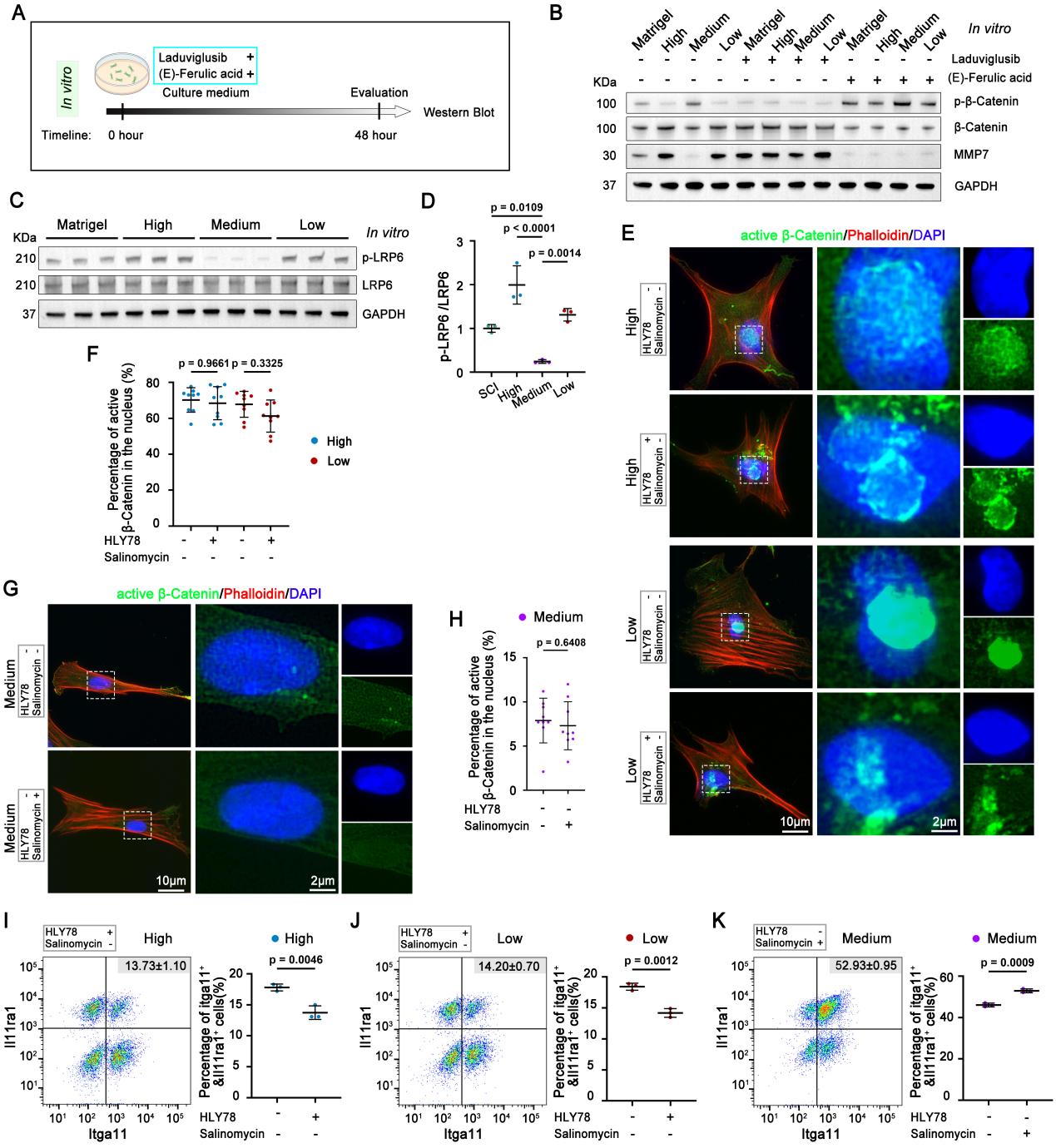


**Figure S13 (related to Figure 6). LRP6-Wnt/β-Catenin-MMP7 signaling axis responded to mechanotransduction.**

1. Schematic of the experimental design to investigate the relationship between active β-catenin and MMP7 *in vitro*. (B) The representative chopped Western blot images showing the expression level of phosphorylated β-catenin, β-catenin and MMP7 *in vitro* following the intervention with Laduviglusib and (E)-Ferulic acid respectively. (C-D) The representative chopped Western blot images (C) and quantitation (D) showing the expression of LRP6 and phosphorylated LRP6 proteins *in vitro.* N = 3 samples. (E) Representative multichannel immunostaining images showing the expression active β-catenin and phalloidin in the migrating fibroblasts in High and Low hydrogel group following intervention with HLY78. The white boxed regions were higher magnifications of the active β-catenin signal and DAPI, which were presented in separate channels. (F) The quantifications of the proportion of active β-catenin entering nucleus following the intervention with HLY78 in High and Low hydrogel group. N = 9 cells. (G) Representative multichannel immunostaining images showing the expression active β-catenin and phalloidin in the migrating fibroblasts in Medium hydrogel group following intervention with Salinomycin. The white boxed regions were higher magnifications of the active β-catenin signal and DAPI, which were presented in separate channels. (H) The quantifications of the proportion of active β-catenin entering nucleus following the intervention with Salinomycin in Medium hydrogel group. N = 9 cells. (I-J) Flow cytometric analysis of Itgall^+^/Il11ra1^+^ fibroblasts *in vitro* in High hydrogel group (I) and Low hydrogel group (J) following intervention with HLY78. N = 3 samples. (K) Flow cytometric analysis of Itgall^+^/Il11ra1^+^ fibroblasts *in vitro* in Medium hydrogel group following intervention with Salinomycin. N = 3 samples, respectively. Data were shown as the mean ± SD; P values were determined by two-tailed one-way ANOVA with Tukey’s multiple-comparisons test or two-tailed paired t-tests.


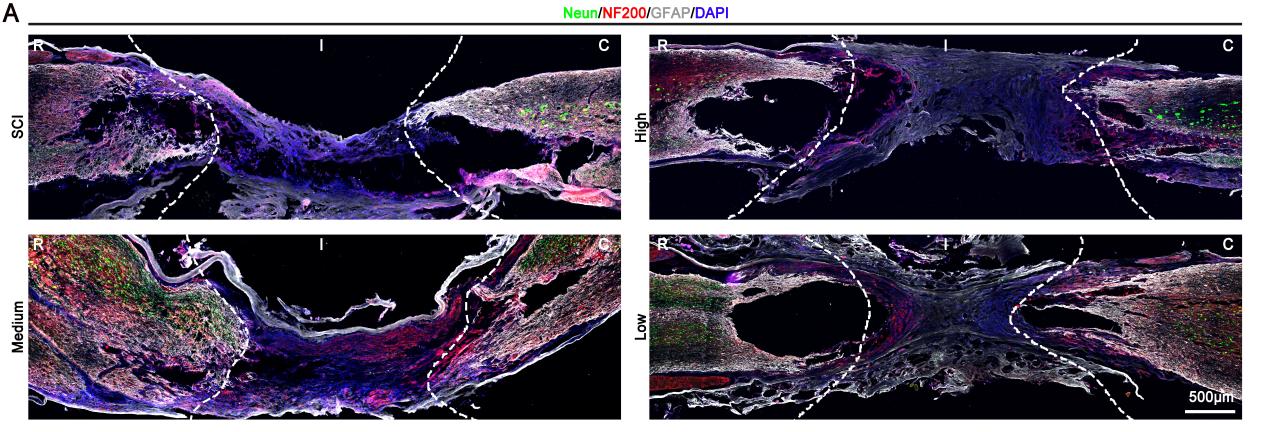


**Figure S14 (related to Figure 7). Promotion of Axonal Regeneration at 4 Wpi.**

1. Representative multichannel immunostaining images revealing the expression of NF200, Neun, and GFAP in the longitudinal sections of spinal cord at 4 wpi.

Table S1. Information of antibodies for immunofluorescence utilized in the study.

| Name | abbreviation | host | dilution | company |
| --- | --- | --- | --- | --- |
| Anti- glial fibrillary acidic protein | GFAP | Chicken | 1:500 | Abcam |
| Anti-Laminin | Ln | Rabbit | 1:500 | Abcam |
| Anti-Collagen, type IV | Col IV | Rabbit | 1:500 | Abcam |
| Anti-Collagen, type I | Col I | Rabbit | 1:500 | Abcam |
| Anti-Fibronectin | Fn | Rabbit | 1:500 | Abcam |
| Anti-Collagen, type I, alpha 1 | Col1a1 | Mouse | 1:2000 | Sigma |
| Anti- PDGF R beta | Anti-PDGFRβ | Goat | 1:200 | R&D systems |
| Anti- Integrin Subunit Alpha 11 | Itga11 | Goat | 1:500 | Santa Cruz Biotechnology |
| Anti-Il11 Receptor, alpha 1 | II11ra1 | rabbit |  | Biorbyt |
| Anti-Phalloidin | Phalloidin |  | 1:200 | Solarbio |
| Anti-detyrosinated tubulin | DetyrTub | Rabbit | 1:500 | Millipore |
| Anti-Vinculin | Vinculin | Rabbit | 1:500 | Abcam |
| Anti-beta-Catenin | β-Catenin | Rabbit | 1:500 | Abcam |
| Anti-Neurofilament Heavy Chain | NF200 | Chicken | 1:1000 | Abcam |
| Anti-Neun | Neun | Rabbit | 1:500 | Abcam |
| Anti-MAP2 | MAP2 | Mouse | 1:500 | Millipore |
| Anti-Synapsin Ⅰ | SYN | Mouse | 1:200 | Invitrogen |
| Anti-Synapsin Ⅰ | SYN | Rabbit | 1:500 | Servicebio |
| Anti-vGLUT | vGLUT | Rabbit | 1:500 | Synaptic Systems |
| Anti-vGAT | vGAT | Rabbit | 1:1000 | Synaptic Systems |
| Anti-mouse IgG 647 |  | donkey | 1:500 | Abcam |
| Anti-rabbit IgG 555 |  | donkey | 1:500 | Abcam |
| Anti-chicken IgG 488 |  | donkey | 1:500 | Abcam |
| Anti-goat IgG 488 |  | donkey | 1:500 | Abcam |

Table S2. Information of antibodies for Western blot utilized in the study.

| Name | abbreviation | host | dilution | company |
| --- | --- | --- | --- | --- |
| Anti-detyrosinated tubulin | DetyrTub | Rabbit | 1:3000 | Millipore |
| Anti-Matrix Metalloproteinase 7 | MMP7 | Rabbit | 1:2000 | Origene |
| Anti-phospho-β-Catenin (Ser33/37/Thr41) | p-β-Catenin | Rabbit | 1:5000 | Cell Signaling Technology |
| Anti-β-Catenin | β-Catenin | Rabbit | 1:5000 | Cell Signaling Technology |
| Anti-phospho-LRP6 (Ser1490) | p-LRP6 | Rabbit | 1:3000 | Cell Signaling Technology |
| Anti-LRP6 | LRP6 | Rabbit | 1:5000 | Cell Signaling Technology |
| Anti-β3-tubulin | Tuj1 | Rabbit | 1:5000 | Cell Signaling Technology |
| Anti-Neurofilament Heavy Chain | NF200 | Chicken | 1:10000 | Abcam |
| Anti-Vinculin | Vinculin | Rabbit | 1:3000 | Abcam |
| Anti-GAPDH | GAPDH | Mouse | 1:10000 | Abcam |
| IgG, HRP-linked Antibody |  | Rabbit | 1:5000 | Cell Signaling Technology |
| IgG, HRP-linked Antibody |  | Mouse | 1:5000 | Cell Signaling Technology |
| IgG, HRP-linked Antibody |  | Chicken | 1:5000 | Cell Signaling Technology |
